# Kinematic and aerodynamic modeling of flexible wings with wing root adjustment for flapping wing micro aerial vehicles

**DOI:** 10.1038/s41598-026-40582-8

**Published:** 2026-03-02

**Authors:** Ziming Liu, Xiaoya Zhang, Zihao Wang, Ruiqi Ye

**Affiliations:** 1 School of Information and Electronics, Beijing Institute of Technology, Beijing, 100081 China; 2 Chinese Institute of Electronics, Beijing, 100036 China; 3 Institute of Information Engineering, Chinese Academy of Sciences, Beijing, 100085 China; 4 Innovation Academy for Microsatellites, Chinese Academy of Sciences, Shanghai, 201304 China; 5 School of Aerospace Engineering, Beijing Institute of Technology, Beijing , 100081 China

**Keywords:** Engineering, Physics

## Abstract

In the field of insect-like flapping-wing micro aerial vehicle (FWMAV) research, this study focuses on a specific type of FWMAV that is tailless and possesses hovering capabilities. The most common method for generating aerodynamic moment in these vehicles is wing-root adjustment. By varying the twist of the wing root, an uneven or asymmetric aerodynamic distribution is created, which generates control moments. This study develops a kinematic model of flexible wings based on wing-root adjustment for aerodynamic design of insect-like FWMAVs and proposes a new aerodynamic simulation method based on multi-rigid-plane fitting to validate the kinematic model. The complete modeling framework for insect-like FWMAVs is first established, which includes the drive system transmission model, the kinematic model of wing motion under wing-root adjustment, an aerodynamic model based on a quasi-steady method, and a multi-rigid-body dynamics model. Next, the kinematics of the flexible wings are divided into three components: the relaxation phase angle equation, the wing motion equation, and the amplitude calibration equation. Then, multi-rigid plane fitting is carried out for the flexible wings based on the topological structure of the wing vein network. Taking the wing kinematics model as input, aerodynamic simulation is conducted by combining with the blade element theory. Finally, the aerodynamic simulation results are compared with results from single-plane simulations and experimental measurements. The results show that the kinematic model of the flexible wings under wing-root adjustment and the multi-plane aerodynamic simulation method are highly effective in predicting the variation of aerodynamic moment in flexible wings. The estimation error for lift does not exceed 20%, providing valuable guidance for subsequent controller design.

**Supplementary Information:**

The online version contains supplementary material available at 10.1038/s41598-026-40582-8.

## Introduction

Insect-like FWMAVs possess unique advantages such as high maneuverability, high energy efficiency, and the ability to hover, making them a focal point in the field of bioinspired aircraft research. This study specifically focuses on tailless, dual-wing configurations of insect-like FWMAVs. Numerous research efforts around the world have been dedicated to developing prototype flapping-wing vehicles with this configuration^1–13^. Notable examples include AeroVironment’s Nano Hummingbird^1^ (although inspired by hummingbirds, its flight mechanism closely resembles that of insects), the Colibri^2^, and the KUBeetle series by Phan’s team, KUBeetle^3^, KUBeetle-S^4^, and foldable-wing designs^5^. In achieving stable flight, insect-like FWMAVs face three core challenges:

First, their aerodynamic mechanisms are highly complex, involving phenomena such as delayed stall^14–17^, wake capture^18–20^, and the clap-and-fling (C-F) mechanism^21–23^.

Second, the attitude control of insect-like flapping-wing aircraft faces significant technical challenges. Insects achieve precise flight through highly complex wing kinematic mechanisms evolved over time, including instantaneous direction adjustment and three-dimensional trajectory control^24–30^, coupled with various biological sensing systems^31–34^. When artificial biomimetic systems replicate such natural mechanisms, they will encounter difficulties in three-dimensional flapping design of the wings and integration of micro sensing-control systems.

Third, the inherently low Reynolds number of flapping-wing systems^35^ results in unsteady aerodynamic effects during hovering^36,37^, which greatly increases the complexity of achieving autonomous stable control^38–40^.

Therefore, during prototype development, the modeling and co-optimization of wing kinematics and aerodynamics are not only key technical bottlenecks but also critical to enhancing the overall bio-mimetic performance of these aerial vehicles.

Current research on the kinematic and aerodynamic modeling of flapping-wing vehicles faces several critical limitations:

In the field of kinematic modeling, the existing studies^41–49^ on wing surface modeling are mainly based on two assumptions: two-dimensional rigid wings and three-dimensional flexible wings. The former is represented by Fan, Walker, Sane and Lehmann^41–44^. By establishing a two-dimensional rigid wing model, the flapping motion angle is simplified as a function representation based on Fourier series. Anno and Liu^45,46^ also adopt this assumption. The latter category focuses on the elastic deformation characteristics of the wing, with Du^47^, Tuncer, and Berman^48,49^ developing 3D kinematic models from the perspectives of wing spar flexibility and rigid-flexible coupling, respectively. However, both modeling approaches fail to address the three-dimensional kinematics under wing-root adjustment, a key mechanism responsible for generating control moments during free flight of actual prototypes^3,50,51^. This gap in kinematic modeling leads directly to limitations in aerodynamic modeling: the current kinematic frameworks do not adequately capture how wing-root movements affect the instantaneous flapping states of flexible wings, resulting in incomplete or inaccurate input parameters for aerodynamic simulations. Therefore, developing a three-dimensional kinematic model that can characterize the coupled effects of spatial wing-root adjustment and flexible wing deformation has become a crucial prerequisite for the aerodynamic design of wing-root-actuated FWMAVs.

In the field of aerodynamic modeling, the most commonly used approaches include experimental measurements^52^, CFD-FSI (Computational Fluid Dynamics and Fluid-Structure Interaction) simulations^47,53,54^, and blade element theory based on quasi-steady aerodynamic models^42,55^. Among these, experimental measurements offer the highest accuracy but it is difficult to provide theoretical guidance for the aerodynamic design of wings. CFD-FSI method^47^ predicts the laws of fluid motion by numerically solving fluid dynamics equations, offering relatively high prediction accuracy. However, it suffers from high computational complexity, long computation times, and slow iteration cycles. In contrast, blade element theory based on quasi-steady assumptions offers high computational efficiency^23^, but its aerodynamic estimations for flexible wings often carry large errors. Moreover, it is difficult to combine the wing with continuous flexible deformation under wing-root adjustment with it for aerodynamic estimation. As a result, current aerodynamic modeling approaches for flexible wings lack a theoretical framework capable of efficiently quantifying the coupled interactions among wing-root adjustment, flexible deformation, and aerodynamic response. This limitation hampers rapid iteration in the wing aerodynamic design. Therefore, it is necessary to construct an efficient computational tool for aerodynamic simulation of three-dimensional flexible wings under wing-root adjustment, to provide effective aerodynamic data for control algorithms.

To address the challenges associated with the missing kinematic modeling of three-dimensional flexible wing movements and the difficulty in quantifying aerodynamic effects under the wing-root adjustment of insect-like FWMAVs, this study presents an integrated modeling framework that considers both wing kinematics and aerodynamics under wing-root adjustment. Furthermore, a flexible wing surface rigidity partitioning method based on the topology of wing veins is proposed. This method divides the rigid wing surface elements according to the orientation of the wing veins. Subsequently, based on the quasi-steady assumption, the dynamic deformation of the wing surface is discretized into two-dimensional rigid planar elements for aerodynamic load calculations, with the kinematic model of the wing serving as input. Finally, experiments are conducted to analyze the relationship between the wing flapping frequency and lift and aerodynamic moments under pitch and roll wing-root adjustments. The experimental results are compared with the model predictions. The key points are summarized as follows:

1. The proposed model supplements the kinematic model of hovering-type flapping-wing vehicles under wing-root adjustment. By incorporating the topology of wing veins to partition rigid wing surface elements for aerodynamic simulation, a comprehensive framework for flexible wing kinematics and aerodynamics is established. This provides a research approach for flapping-wing vehicles and lays the foundation for future precise aerodynamic predictions of flexible wings.
2. The model accurately predicts the influence of wing-root adjustments on aerodynamic forces and moments under fixed flapping frequencies, and quantifies these effects through experimental comparisons. The predicted aerodynamic lift achieves an accuracy of over 80 %, offering essential insights into the impact of wing-root adjustments on lift. This will serve as a focal point for subsequent research.

## Related work

### Coordinate system representation and wing description

**Fig. 1:**
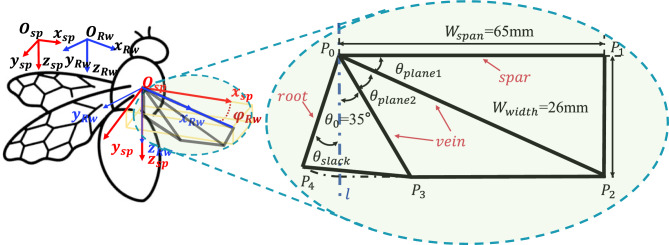
Coordinate system definition and wing design diagram.

During the development of insect-like FWMAV, wing design requires consideration of structural configuration, material properties, kinematic modeling and aerodynamic characteristic analysis. Currently, research teams that have achieved hover flight in FWMAVs all adopt the technical scheme of a rope-driven structure combined with radial wings^1,3,56^, in which the wing spars and veins converge at the origin of the wing root in a radial pattern. This study also takes the radial wing as the research object, and its geometric configuration is shown in the right half of Fig. 1. The structure includes four units: wing spar, wing vein, wing root, and wing membrane. The geometric parameters of each unit are defined as follows: the wingspan length $W_{span}$ and chord length $W_{width}$ form the basic dimensions; the first plane angle $\theta _{plane1}$ is the angle between the first wing vein and the wing spar, and the second plane angle $\theta _{plane2}$ is the angle between the second wing vein and the first wing vein; points $P_{0}-P_{4}$ correspond to the wing root origin and key position nodes on the outer edge. Notably, the slack angle $\theta _{slack}$ is defined as the angle between the wing root $P_{0}P_{4}$ and the vertical line *l* of the wing spar. This parameter has a significant impact on control moment generation, and its mechanism will be discussed in detail in Section 4.3.

The left half of Fig. 1 illustrates the coordinate system architecture for the right wing of the insect-like FWMAV. The subscript *sp* denotes the stroke plane coordinate system, and *Rw* denotes the right-wing coordinate system. The left and right wings are completely symmetric. For the stroke plane coordinate system $Oxyz_{sp}$ the origin $O_{sp}$ is defined at the wing root. The $Oz_{sp}$ axis points downward, the $Ox_{sp}$ axis aligns with the positive direction of the vehicle’s motion, and the $Oy_{sp}$ axis is perpendicular to the $Ozx_{sp}$ plane, following the right-hand rule. The wing coordinate system $Oxyz_{Rw}$ is centered at the same wing root point $O_{Rw}$, where the $Ox_{Rw}$ axis extends along the wing spar, the $Oz_{Rw}$ axis is collinear with the $Oz_{sp}$ axis, and the $Oy_{Rw}$ axis is orthogonal to the $Oxz_{Rw}$ plane, also following the right-hand rule. When the wing generates a flapping angle $\varphi _{Rw}$, the stroke plane coordinate system rotates around the $Oz_{sp}$ axis by $\varphi _{Rw}$ to obtain the wing coordinate system. The coordinate transformation between the two systems is described by the rotation matrix in formula 1. The right half of Fig. 1 shows the wing surface with five edge points: $P_{0}$, $P_{1}$, $P_{2}$, $P_{3}$, and $P_{4}$, representing the three-dimensional wing surface $S_{01234}$.

1 $$\begin{aligned} R_{s p \rightarrow R w} = \begin{bmatrix} c o s \varphi _{R w} & - s i n \varphi _{R w} & 0 \\ s i n \varphi _{R w} & c o s \varphi _{R w} & 0 \\ 0 & 0 & 1 \end{bmatrix} \end{aligned}$$

**Fig. 2:**
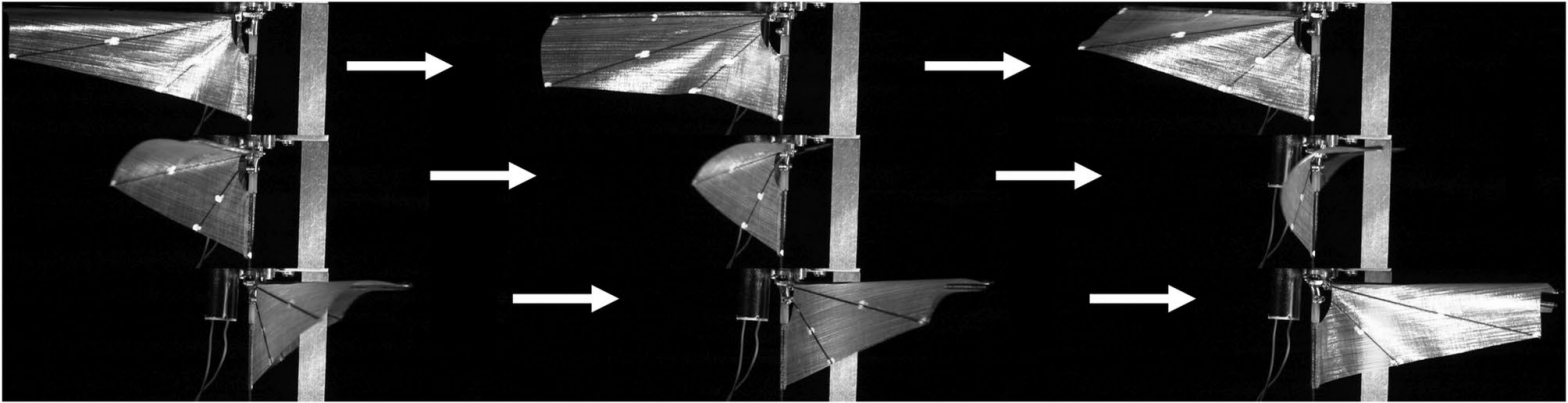
The flapping process of the wing captured by a high-speed camera.

The high-speed camera footage in Fig. 2 captures the wing’s flapping process. During each cycle, the relative motion among the wing spar, the wing veins, and the wing root, together with their constraints on the wing membrane, causes the entire flexible membrane to behave as three dynamically changing planes $S_{012}$, $S_{023}$, and $S_{034}$ By analytically relating the kinematic constraints of these three planar elements, one can construct a mathematical model that describes both the wing’s motion and its deformation. Depending on whether external inputs are applied, this kinematic modeling problem is divided into two modes: the equilibrium mode and the wing-root adjustment mode. In the equilibrium mode, the wing root does not twist during flapping, whereas in the wing-root adjustment mode, the wing root undergoes axial torsion around its origin.

### Model framework

**Fig. 3:**
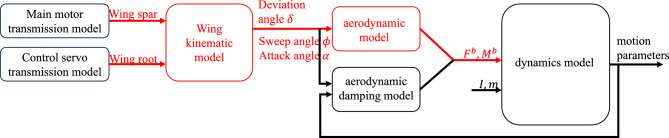
Modeling framework of insect-like FWMAV.

The modeling framework for the insect-like FWMAV is illustrated in Fig. 3. The motor-driven transmission mechanism serves as the input to the kinematic model, driving the periodic flapping motion of the wings. The output of the kinematic model consists of the wing motion parameters, which are then used as inputs for the aerodynamic model. The aerodynamic model subsequently outputs the aerodynamic forces and moments to the dynamics model. The components highlighted in red in the figure represent the key focus areas of this study.

### Wing-root adjustment mode

The control moment generation mechanism of insect-like FWMAV mainly includes three technical paths:

1. Wing frequency and wing amplitude adjustment: Suitable for direct motor-driven configurations, it changes the flapping frequency and amplitude of wings by adjusting the rotation direction and speed of the drive motor, thereby forming an asymmetric aerodynamic load distribution.
2. Flapping plane adjustment: Using a linkage mechanism to dynamically adjust the spatial orientation of the flapping plane, control moment is generated by changing the direction of the aerodynamic resultant force vector. The adjustment of the flapping plane inclination can be considered from two perspectives: dividing the aircraft into upper and lower parts, where only inclining the upper part (i.e., the flapping plane) is called flapping plane adjustment, while adjusting the center of gravity of the lower part to induce flapping plane inclination is also referred to as center of gravity position adjustment.
3. Active wing-root adjustment: With the help of a servo-driven mechanism, the wing root undergoes axial torsion around its origin, inducing asymmetric flapping of the left and right wings to generate control moment.

**Fig. 4:**
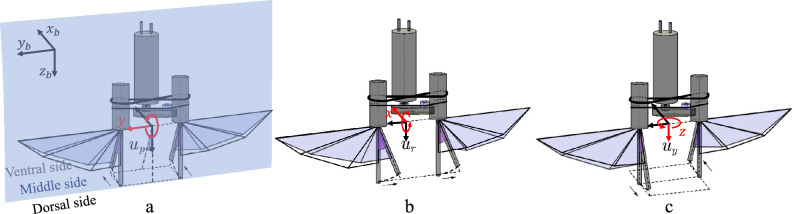
Schematic diagram of the multi-axis control moments generation mechanism of FWMAV.

The three-axis control moments generated by the wing-root adjustment method can be decoupled, which is superior to the first two technical routes in both control scheme design and structural design. This study focuses on the third technical path, generating target control moments through precise regulation of the wing root torsion angle, and its action mechanism is shown in Fig. 4.

As shown in Fig. 4a for the pitch control moment generation mechanism, the flight controller generates a rudder angle command $u_{p}$ by driving the left and right wing roots to synchronously twist around the aircraft body *y*-axis. When the wing root twists forward, the tension of the wing membrane increases during the flapping process from the dorsal side to the middle side of the aircraft, resulting in an increase in the average attack angle, while the tension in the area from the middle side to the ventral side relaxes, causing the attack angle to decrease; the reverse twist shows the opposite law. Within the range of $\left[ 0 \sim 45^{\circ }\right]$ for the attack angle, the lift coefficient is positively correlated with the change of the attack angle^57^. Therefore, the asymmetric distribution of the attack angle in the upward and downward flapping strokes forms a lift gradient difference, thereby generating a pitch moment around the aircraft body *y*-axis.

Figure 4b shows the roll control mechanism. The left and right wing roots synchronously twist around the aircraft body *x*-axis to generate a rudder angle $u_{r}$. When twisting to the right, the tension of the left wing membrane increases, leading to an increase in the lift, and the right wing experiences lift attenuation due to tension relaxation, thus forming a roll moment around the aircraft body *x*-axis.

The yaw control shown in Fig. 4c is based on the aerodynamic drag difference mechanism. The wing roots twist in opposite directions around the aircraft body *x*-axis to generate a rudder angle $u_{y}$. The asymmetric flapping attack angle distribution leads to an asymmetric aerodynamic drag distribution, thereby generating a yaw moment around the aircraft body *z*-axis.

## Method

### The kinematics of wing flapping in equilibrium mode

The wing trajectory equations are given as shown in formulas 2 and 3:

2 $$\begin{aligned} & F:\left\{ \begin{array}{l} F_{0}=f_{0}\left( f_{R W}\right) \\ F_{1}=f_{1}\left( f_{R W}\right) \\ F_{2}=f_{2}\left( f_{R W}\right) \end{array}\right. \end{aligned}$$

3 $$\begin{aligned} & G:\left\{ \begin{array}{c} \varphi _{R w}=g_{0}\left( t, F_{0}\right) \\ \varphi _{R w v_{1}}=g_{1}\left( t, F_{1}\right) \\ \varphi _{R w v_{2}}=g_{2}\left( t, F_{2}\right) \end{array}\right. \end{aligned}$$

The set of amplitude calibration equations *F* in formula 2, comprises three functions $f_{0}$, $f_{1}$, and $f_{2}$. These functions compensate for the nonlinear influence of flapping frequency on the kinematic parameters of the wing spar and veins, where $F_{0}$, $F_{1}$, and $F_{2}$ represent the maximum flapping amplitudes of the spar and the two veins. The wing motion equations *G* in formula 3, describe the dynamic deformation of the wing and involve three key flapping angles: $\varphi _{Rw}$, $\varphi _{Rwv_{1}}$, and $\varphi _{Rwv_{2}}$. These angles are defined as the instantaneous included angles between the stroke plane $Oyz_{sp}$ and the wing spar, the first vein, and the second vein.

**Fig. 5:**
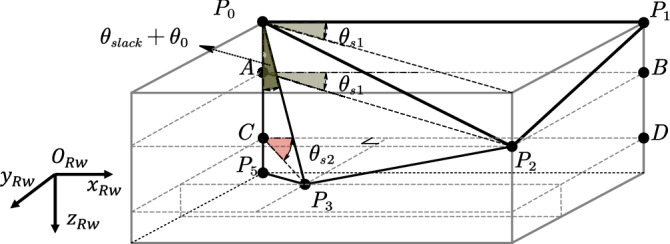
Schematic diagram of the geometric relationship of relaxation phase angles.

Angle $\theta _{s1}$ refers to the included angle between the projection of wing vein $P_{0}P_{2}$ onto the stroke plane and the wing spar $P_{0}P_{1}$, as denoted by $\angle P_{2}AB$ in Fig. 5. Herein, $AP_{2}$ represents the projection of $P_{0}P_{2}$, and *AB* is parallel to $P_{0}P_{1}$, thus $\angle P_{2}AB$ corresponds to $\theta _{s1}$.

Angle $\theta _{s2}$ denotes the included angle between the projection of wing vein $P_{0}P_{3}$ onto the stroke plane and the wing spar $P_{0}P_{1}$, as indicated by $\angle P_{3}CD$ in Fig. 5. Specifically, $CP_{3}$ is the projection of $P_{0}P_{3}$, and *CD* is parallel to $P_{0}P_{1}$, meaning $\angle P_{3}CD$ corresponds to $\theta _{s2}$.

Both $\theta _{s1}$ and $\theta _{s2}$ are defined as the relaxation phase angles (RPAs) of the wing, which quantify the degree of passive deformation of the wing.

In previous studies, flapping wings are often modeled as rigid planar surfaces described by three rotational angles: sweep, deviation, and inclination^58^. Among these, the sweep angle is the dominant motion. While many studies simplify this trajectory to a pure sinusoid^11^, biological observations by Berman et al.^48^—based on data from fruit flies, hawkmoths^38^, and robotic experiments^44^—established a general form for the sweep angle. Their model utilizes a shape parameter *K* to describe the transition between sinusoidal $(K\rightarrow 0)$ and triangular $(K\rightarrow 1)$ waveforms.

Inspired by Berman’s research, we propose a modified kinematic model designed to address the unique wing root adjustment mechanism in the present study (see Section 4.3). Neither the standard sinusoidal model nor the general form proposed by Berman can explicitly quantify the wing deformation induced by wing root adjustment. To address this limitation, we have established a piecewise continuous function g(t), as shown in formula 4. The kinematic equation can be expressed as the linear superposition of uniform-angular and sinusoidal angular motions. Its analytical form introduces five key parameters: dynamic amplitude coefficient *A*, static amplitude offset *B*, flapping period *T*, constant angular speed *k*, sinusoidal angular frequency $\omega$. To ensure smooth transitions at the phase-switch instants $t_{1}$ through $t_{4}$, the trajectory must satisfy $C^{1}$ continuity, enforced via the first-derivative continuity condition in formula 5. The nonlinearity parameter $\delta \in [0,1]$ is defined to quantify the proportion of the sinusoidal component within each flapping period *T* in formula 4, expressed as $\frac{t_{2} - t_{1} + t_{4} - t_{3}}{T}$. When $\delta$=1 the motion trajectory approaches a standard sine function; when $\delta$=0, it degenerates into an ideal triangular wave function. The specific values are shown in Experimental Section 6.1.

4 $$\begin{aligned} & g_{0}(t) = \left\{ \begin{array}{l} \begin{array}{l} {kt} \\ {Acos\omega \left( {t - 0.25T} \right) + B} \end{array} \\ \begin{array}{l} {- k\left( {t - 0.5T} \right) } \\ {Acos\omega \left( {t - 0.25T} \right) - B} \\ {k\left( {t - T} \right) } \end{array} \end{array}~~~~~\begin{array}{l} \begin{array}{l} {0 \le t< t_{1}} \\ {t_{1} \le t< t_{2}} \end{array} \\ \begin{array}{l} {t_{2} \le t< t_{3}} \\ {t_{3} \le t< t_{4}} \\ {t_{4} \le t < T} \end{array} \end{array} \right. \end{aligned}$$

5 $$\begin{aligned} & \left\{ \begin{matrix} {A + B = 0.5F_{0}} \\ {B = k\left( {0.25T - \delta } \right) } \\ {k = \frac{A\pi }{2\delta }} \end{matrix} \right. \end{aligned}$$

**Fig. 6:**
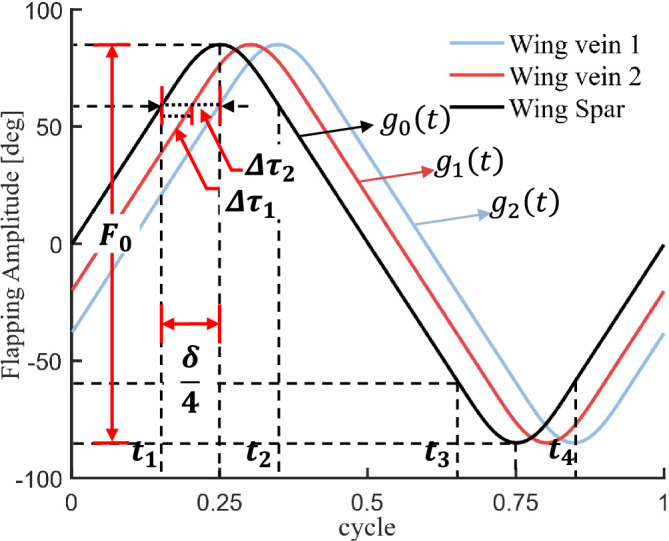
Time-domain characteristic curves of the wing kinematic equations.

Figure 6 shows the simulation results of formulas 4 and 5, where the kinematic equations of wing veins 1 and 2 generate phase offsets $\Delta \tau _{1}$ and $\Delta \tau _{2}$ respectively based on $g_{0}\left( t\right)$, and their mathematical relationships are described by formula 6.

6 $$\begin{aligned} \left\{ \begin{matrix} {g_{1}(t) = g_{0}\left( {t - \Delta \tau _{1}} \right) ~~~~~~~~~\Delta \tau _{1} = \frac{\theta _{s1}}{k}} \\ {g_{2}(t) = g_{0}\left( {t - \Delta \tau _{2}} \right) ~~~~~~~~~\Delta \tau _{2} = \frac{\theta _{s2}}{k}} \end{matrix} \right. \end{aligned}$$

For the amplitude calibration equation *F*, the insect-like FWMAV in this study adopts a rope-driven structure, and the technology is derived from the team’s previous work^59^. It has been confirmed in previous studies that during the periodic flapping of the wings, the elastic deformation of the polyethylene (PE) transmission rope at different flapping frequencies will lead to significant changes in the flapping amplitude, which must be dynamically compensated by the calibration model shown in formula 7. ${Am}_{0}$ and ${Bm}_{0}$ represent the amplitude calibration parameters of the wing spar, which can be obtained through experiments shown in Experimental Section 6.2.

7 $$\begin{aligned} F_{0} = {Am}_{0}f + {Bm}_{0} \end{aligned}$$

### The coordinates of the flapping point in equilibrium mode

**Fig. 7:**
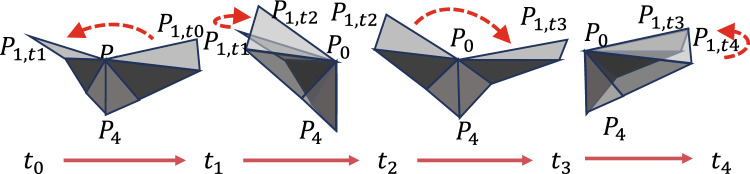
The complete flapping phase of the wing in a single cycle.

As shown in Fig. 7, the complete flapping phase $\left( t_{0}\rightarrow t_{4} \right)$ of the wing within a single cycle includes four stages:

Stage I: $\left( t_{0}\rightarrow t_{1} \right)$ Downstroke process (uniform angular motion, wing surface maintains quasi-rigid characteristics)

Stage II: $\left( t_{1}\rightarrow t_{2} \right)$ Flipping process (dynamic deformation)

Stage III: $\left( t_{2}\rightarrow t_{3} \right)$ Upstroke process (same as $t_{0}\rightarrow t_{1}$)

Stage IV: $\left( t_{3}\rightarrow t_{4} \right)$ Secondary flipping process (symmetric to stage $t_{1}\rightarrow t_{2}$)

The kinematic analysis of the Downstroke/upstroke process (Stage I and Stage III) is conducted in the stroke plane coordinate system $Oxyz_{sp}$ Based on the constraint condition of three-plane area conservation, the spatial coordinates of each point are defined by formula 8.

8 $$\begin{aligned} \left\{ \begin{matrix} {P_{0}\left( {0,0,0} \right) } \\ {P_{1}\left( {x_{1},y_{1},z_{1}} \right) } \\ {P_{2}\left( {x_{2},y_{2},z_{2}} \right) } \\ {P_{3}\left( {x_{3},y_{3},z_{3}} \right) } \\ {P_{4}\left( {0,0,W_{width}} \right) } \end{matrix} \right. \end{aligned}$$

The expressions for the lengths of each side of the wing are shown in formula 9.

9 $$\begin{aligned} \left\{ \begin{array}{l} \begin{array}{l} {\left| {P_{0}P_{1}} \right| = W_{span} = 65mm} \\ {\left| {P_{1}P_{2}} \right| = W_{width} = 28mm} \\ {\left| {P_{0}P_{4}} \right| = W_{width} = 28mm} \end{array} \\ \begin{array}{l} {\left| {P_{0}P_{3}} \right| = \frac{W_{width}}{cos\left( \theta _{0} \right) }} \\ {\left| {P_{0}P_{2}} \right| = \sqrt{\left| {P_{0}P_{1}} \right| ^{2} + \left| {P_{1}P_{2}} \right| ^{2}}} \\ {\left| {P_{2}P_{3}} \right| = W_{span} - \left| {P_{0}P_{3}} \right| sin\left( \theta _{0} \right) } \end{array} \\ {\left| {P_{3}P_{4}} \right| ^{2} = W_{width}^{2} + \left( \frac{W_{width}}{cos\left( \theta _{0} \right) } \right) ^{2} - 2 \cdot W_{width}\frac{W_{width}}{cos\left( \theta _{0} \right) }cos\left( {\theta _{slack} + \theta _{0}} \right) } \end{array} \right. \end{aligned}$$

For each outer edge point, the following derivation process is carried out:

For point $P_{1}\left( {x_{1},y_{1},z_{1}} \right)$, there is formula 10:

10 $$\begin{aligned} P_{1}:\left\{ \begin{matrix} {x_{1} = W_{span}si{n\left( \varphi _{Rw} \right) }} \\ {y_{1} = W_{span}cos\left( \varphi _{Rw} \right) } \\ {z_{1} = 0} \end{matrix} \right. \end{aligned}$$

For point $P_{2}\left( {x_{2},y_{2},z_{2}} \right)$, it is described by formula 11, where the geometric meaning of the vector $\overrightarrow{AP_{2}}$ is shown in Fig. 5 as the projection component of the vector $\overrightarrow{P_{0}P_{2}}$ in the $Oxy_{sp}$ plane.

11 $$\begin{aligned} P_{2}:\left\{ \begin{matrix} {x_{2}^{2} + y_{2}^{2} + z_{2}^{2} = \left| {P_{0}P_{2}} \right| ^{2}} \\ {\left( {x_{2} - x_{1}} \right) ^{2} + \left( {y_{2} - y_{1}} \right) ^{2} + \left( {z_{2} - z_{1}} \right) ^{2} = \left| {P_{1}P_{2}} \right| ^{2}} \\ {\frac{\overrightarrow{AP_{2}} \cdot \overrightarrow{P_{0}P_{1}}}{\left| {AP_{2}} \right| \left| {P_{0}P_{1}} \right| } = cos\left( \theta _{s1} \right) } \end{matrix} \right. \end{aligned}$$

For point $P_{3}\left( {x_{3},y_{3},z_{3}} \right)$, it is described by formula 12.

12 $$\begin{aligned} P_{3}:\left\{ \begin{matrix} {x_{3}^{2} + y_{3}^{2} + z_{3}^{2} = \left| {P_{0}P_{3}} \right| ^{2}} \\ {x_{3}^{2} + y_{3}^{2} + \left( {z_{3} - W_{width}} \right) ^{2} = \left| {P_{3}P_{4}} \right| ^{2}} \\ {\frac{\overrightarrow{CP_{3}} \cdot \overrightarrow{P_{0}P_{1}}}{\left| {CP_{3}} \right| \left| {P_{0}P_{1}} \right| } = cos\left( \theta _{s2} \right) } \end{matrix} \right. \end{aligned}$$

The kinematic analysis of the wing flipping process (Stage II and Stage IV) is shown in formula 13. The three-plane area conservation condition fails, because $\left| {P_{1}P_{2}} \right|$, $\left| {P_{2}P_{3}} \right|$, and $\left| {P_{3}P_{4}} \right|$ exhibit time-varying characteristics, leading to an infinite number of solution sets in formula 13 due to rank deficiency. A constraint condition is established: the projection displacements of wing veins $P_{0}P_{2}$ and $P_{0}P_{3}$ on the *z*-axis are negligible. Under this constraint, the kinematic model in equilibrium mode has mathematical completeness. For the analysis process of this conclusion, please refer to Experimental Section 6.3.

13 $$\begin{aligned} \left\{ \begin{array}{l} \begin{array}{l} {\left| {P_{0}P_{2}} \right| = \sqrt{x_{2}^{2} + y_{2}^{2} + z_{2}^{2}}} \\ {\left| {P_{0}P_{3}} \right| = \sqrt{x_{3}^{2} + y_{3}^{2} + z_{3}^{2}}} \end{array} \\ \begin{array}{l} {\frac{\overrightarrow{AP_{2}} \cdot \overrightarrow{P_{0}P_{1}}}{\left| {AP_{2}} \right| \left| {P_{0}P_{1}} \right| } = cos\left( \theta _{s1} \right) } \\ {\frac{\overrightarrow{CP_{3}} \cdot \overrightarrow{P_{0}P_{1}}}{\left| {CP_{3}} \right| \left| {P_{0}P_{1}} \right| } = cos\left( \theta _{s2} \right) } \end{array} \end{array} \right. \end{aligned}$$

### The kinematics of wing flapping in wing-root adjustment mode

The wing motion trajectory is fully characterized by formula 14, which includes the wing motion equations *G*, the amplitude calibration equations *F*, and the relaxation phase angle (RPA) equations *N*.

14 $$\begin{aligned} G:\left\{ \begin{matrix} {\varphi _{Rw} = g_{0}\left( {t,F_{0}} \right) } \\ {\varphi _{Rwv_{1}} = g_{1}\left( {t,\theta _{s1},F_{1}} \right) } \\ {\varphi _{Rwv_{2}} = g_{2}\left( {t,\theta _{s2},F_{2}} \right) } \end{matrix} \right. ~~F:\left\{ \begin{matrix} {F_{0} = f_{0}\left( f_{RW} \right) } \\ {F_{1} = f_{1}\left( f_{RW} \right) } \\ {F_{2} = f_{2}\left( f_{RW} \right) } \end{matrix} \right. ~~N:\left\{ \begin{matrix} {\theta _{s1} = n_{1}(u)} \\ {\theta _{s1} = n_{2}(u)} \end{matrix} \right. \end{aligned}$$

The RPA equation *N* comprises two components, $n_{1}$and $n_{2}$, which correspond to the RPAs of veins $P_{0}P_{2}$ and $P_{0}P_{3}$, respectively. Their amplitudes are governed by the slack angle $\theta _{slack}$ and by the rudder quantities $u=\left( u_{p},u_{r},u_{y}\right)$. This function quantifies the degree of passive wing deformation: When $\theta _{slack}=0$, the wing membrane is fully tensioned (its elastic deformation is negligible), and the spar and veins move in perfect synchrony. When $\theta _{slack}>0$, a flapping phase difference occurs between the wing spar and the wing veins, which is defined as the RPA to characterize the deformation of the wing.

**Fig. 8:**
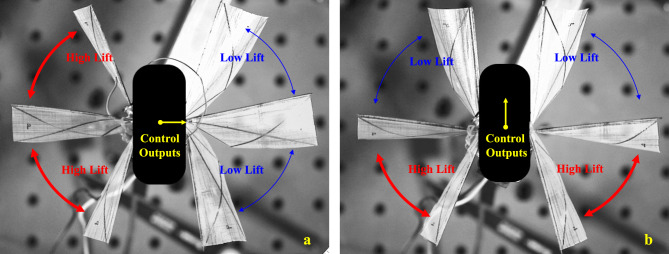
Composite images of wing transient flapping obtained from the top-down perspective of a high-speed camera. (**a**) Roll control mode. (**b**) Pitch control mode.

For the RPA equation *N*, analysis is carried out in two control modes. Figure 8 shows the composite image of the wing’s transient flapping obtained from the top-down perspective of a high-speed camera. Figure 8a displays three instantaneous flapping conditions of the wing under the roll control mode ($u_{r}\ne 0$), showing that the left and right wings exhibit asymmetric deformation characteristics. Figure 8b shows that under the pitch control mode ($u_{p}\ne 0$), the two wings maintain deformation symmetry, but the deformation of a single wing presents gradient changes in the forward/backward strokes.

For the roll control mode, when the rudder is deflected to one side, the deformation of the wing on that side gradually decreases, while the deformation on the other side correspondingly increases. According to the conclusions of the RPA measurement experiment in Experimental Section 6.4, the RPA $\theta$ under the roll control mode exhibits an approximately linear change with the variation of the roll rudder $u_{r}$. The model of the RPA as a function of the roll rudder can be expressed by formula 15, where $\lambda _{L1}$, $\lambda _{L2}$, $\mu _{L1}$, $\mu _{L2}$ are the parameters of the left wing roll RPA equation, and $\lambda _{R1}$, $\lambda _{R2}$, $\mu _{R1}$, $\mu _{R2}$ are the parameters of the right wing roll RPA equation.

15 $$\begin{aligned} \begin{matrix} {Left~wing\left\{ \begin{matrix} {\theta _{s1} = \lambda _{L1}\textbf{u}_{\textbf{r}} + \mu _{L1}} \\ {\theta _{s2} = \lambda _{L2}\textbf{u}_{\textbf{r}} + \mu _{L2}} \end{matrix} \right. } \\ {Right~wing\left\{ \begin{matrix} {\theta _{s1} = \lambda _{R1}\textbf{u}_{\textbf{r}} + \mu _{R1}} \\ {\theta _{s2} = \lambda _{R2}\textbf{u}_{\textbf{r}} + \mu _{R2}} \end{matrix} \right. } \end{matrix} \end{aligned}$$

For the pitch control mode, the RPA exhibits gradient variation characteristics during the downstroke/upstroke process. The RPA is not only regulated by the pitch rudder $u_{p}$, but also affected by the flapping angle $\varphi$. To describe this law and simplify the calculation, a mathematical model of the RPA varying with the flapping angle $\varphi \left( t\right)$ within a single cycle is established: assuming a linear relationship between the RPA $\theta$ and the flapping angle $\varphi$, the analytical expression shown in formula 16 is derived, $m_{1}$, $n_{1}$, $m_{2}$ and $n_{2}$ are the corresponding parameters.

16 $$\begin{aligned} \left\{ \begin{matrix} {\theta _{1} = m_{1}\varphi _{Rwv_{1}} + n_{1}} \\ {\theta _{2} = m_{2}\varphi _{Rwv_{2}} + n_{2}} \end{matrix} \right. \end{aligned}$$

Substituting the boundary points $Q_{1}\left( {\varphi _{Rwv_{1},t_{q1}},\theta _{s1,max}} \right)$, $Q_{2}\left( {\varphi _{Rwv_{1},t_{q2}},\theta _{s1,min}} \right)$, $R_{1}\left( {\varphi _{Rwv_{2},t_{r1}},\theta _{s2,max}} \right)$, and $R_{2}\left( {\varphi _{Rwv_{2},t_{r2}},\theta _{s2,min}} \right)$ of the downstroke/upstroke process into formula 17:

17 $$\begin{aligned} \left\{ \begin{array}{l} \begin{array}{l} {m_{1}\varphi _{Rwv_{1},t_{q1}} + n_{1} = \theta _{s1,max}} \\ {m_{1}\varphi _{Rwv_{1},t_{q2}} + n_{1} = \theta _{s1,min}} \end{array} \\ \begin{array}{l} {m_{2}\varphi _{Rwv_{2},t_{r1}} + n_{2} = \theta _{s2,max}} \\ {m_{2}\varphi _{Rwv_{2},t_{r2}} + n_{2} = \theta _{s2,min}} \end{array} \end{array} \right. \end{aligned}$$

The start and end phase nodes of the down/upstroke process for vein $P_{0}P_{2}$, denoted $Q_{1}$ and $Q_{2}$, correspond to the extrema of the flapping angle $\varphi$ and the RPA $\theta$. Likewise, the boundary points for vein $P_{0}P_{3}$, denoted $R_{1}$ and $R_{2}$, represent the extrema of its RPA and flapping angle.

After confirming the model of the RPA varying with the flapping angle, the next step is to establish a model under pitch control mode. According to the conclusions of Experimental Section 6.4, with the increase of the rudder $u_{p}$, both the maximum RPAs $\left( {\theta _{s1,max},\theta _{s2,max}} \right)$ and the minimum RPAs within a cycle $\left( {\theta _{s1,min},\theta _{s2,min}} \right)$ show approximately linear growth or decline. The established model is shown in formula 18, where $\sigma$ and $\nu$ are the parameters related to the RPA equation under the pitch control mode.

18 $$\begin{aligned} \left\{ \begin{array}{l} \begin{array}{l} {\theta _{s1,max} = \sigma _{s1,max}\textbf{u}_{\textbf{p}} + \nu _{s1,max}} \\ {\theta _{s1,min} = \sigma _{s1,min}\textbf{u}_{\textbf{p}} + \nu _{s1,min}} \end{array} \\ \begin{array}{l} {\theta _{s2,max} = \sigma _{s2,max}\textbf{u}_{\textbf{p}} + \nu _{s2,max}} \\ {\theta _{s2,min} = \sigma _{s2,min}\textbf{u}_{\textbf{p}} + \nu _{s2,min}} \end{array} \end{array} \right. \end{aligned}$$

By combining formulas 16, 17 and 18, the RPA equation *N* under the pitch control mode is shown in formula 19:

19 $$\begin{aligned} \left\{ \begin{matrix} {\theta _{1} = \frac{\left( {\sigma _{s1,max} - \sigma _{s1,min}} \right) \textbf{u}_{\textbf{p}} + \nu _{s1,max} - \nu _{s1,min}}{\varphi _{Rwv_{1},t_{q1}} - \varphi _{Rwv_{1},t_{q2}}}\mathbf {\varphi }_{\textbf{R}\textbf{w}\textbf{v}_{1}} - \frac{\theta _{s1,max}\varphi _{Rwv_{1},t_{q2}} - \theta _{s1,min}\varphi _{Rwv_{1},t_{q1}}}{\varphi _{Rwv_{1},t_{q1}} - \varphi _{Rwv_{1},t_{q2}}}} \\ {\theta _{2} = \frac{\left( {\sigma _{s2,max} - \sigma _{s2,min}} \right) \textbf{u}_{\textbf{p}} + \nu _{s2,max} - \nu _{s2,min}}{\varphi _{Rwv_{2},t_{r1}} - \varphi _{Rwv_{2},t_{r2}}}\mathbf {\varphi }_{\textbf{R}\textbf{w}\textbf{v}_{2}} - \frac{\theta _{s2,max}\varphi _{Rwv_{2},t_{r2}} - \theta _{s2,min}\varphi _{Rwv_{2},t_{r1}}}{\varphi _{Rwv_{2},t_{r1}} - \varphi _{Rwv_{2},t_{r2}}}} \end{matrix} \right. \end{aligned}$$

Next, the wing motion equations *G* are determined. The wing spar motion equation $g_{0}\left( t,F_{0}\right)$ inherits the derivation results from formulas 4 and 5, and the wing vein motion equation takes the equation *N* as input. Formula 19 only gives the RPA during uniform angular motion. It is necessary to use $g_{0}\left( t,F_{0}\right)$ and the measured $\theta _{s1,max}$, $\theta _{s1,min}$, $\theta _{s2,max}$, $\theta _{s2,min}$ to determine the expression of the flapping angle for the linear part of the wing veins, while the nonlinear part is fitted by trigonometric functions.

**Fig. 9:**
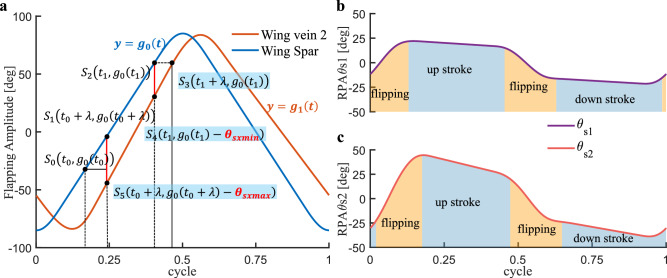
Curves of wing spar and veins motion equations. (**a**) Flapping curves of wing spar and veins under pitch control mode. (**b**) and (**c**) Curves of RPAs $\theta _{s1}$ and $\theta _{s2}$ within a single cycle.

Taking the solution of the wing vein $P_{0}P_{3}$ as an example. The kinematic function is given by formula 20. Figure 9a shows the motion function curves for the wing spar and vein $P_{0}P_{3}$ under pitch control mode. The blue curve represents the motion of the wing spar $g_{0}\left( t,F_{0}\right)$, while the red curve represents the motion of vein $P_{0}P_{3}$ ($g_{2}\left( t,F_{2}\right)$), assuming the maximum flapping amplitudes of both the wing spar and veins are identical.

Given $g_{0}\left( t,F_{0}\right)$, solve for the function $g_{2}=k_{1}t+b_{1}$. First, the interval of the linear part should match the size of the linear interval of $g_{0}\left( t,F_{0}\right)$. As shown in Fig. 9a, the segment from point $S_{0}$ to point $S_{2}$ corresponds to the linear part of $g_{0}$, with the interval $\left[ t_{0},t_{1}\right]$. Suppose the translation of $g_{1}$ relative to $g_{0}$ is $\lambda$. Then, the segment from point $S_{5}$ to point $S_{3}$ corresponds to the linear part of $g_{2}$, with the interval $\left[ t_{0}+\lambda ,t_{1}+\lambda \right]$.

The parameters $\lambda$,*k*1, and *b*1 are determined by the boundary conditions imposed by the RPA. Specifically, the maximum and minimum RPAs $(\theta _{s2,max},\theta _{s2,min})$ correspond to the differences between $g_{0}$ and $g_{2}$ at the start and end of the linear phase (represented by intervals $S_{5}S_{1}$ and $S_{4}S_{2}$ in Fig. 9a). Solving the geometric constraints yielded by these points leads to the analytical solution in Eq. 21.

20 $$\begin{aligned} & g_{2}(t) = \left\{ {\begin{array}{l} \begin{array}{l} {k_{1}t + b_{1}} \\ {A_{1}cos\omega _{1}\left( {t - 0.5\left( {t_{1} + t_{2}} \right) } \right) + B_{1}} \end{array} \\ \begin{array}{l} {k_{2}t + b_{2}} \\ {A_{2}cos\omega _{2}\left( {t - 0.5\left( {t_{3} + t_{4}} \right) + \pi } \right) + B_{2}} \\ {k_{1}\left( {t - T} \right) + b_{1}} \end{array} \end{array}~~~~~\begin{array}{l} \begin{array}{l} {0 \le t< t_{1}} \\ {t_{1} \le t< t_{2}} \end{array} \\ \begin{array}{l} {t_{2} \le t< t_{3}} \\ {t_{3} \le t< t_{4}} \\ {t_{4} \le t < T} \end{array} \end{array}} \right. \end{aligned}$$

21 $$\begin{aligned} & \left\{ \begin{matrix} {k_{1} = - \frac{g_{0}\left( t_{1} \right) - g_{0}\left( {t_{0} + \lambda } \right) + \theta _{s2,max}}{t_{0} - t_{1}}} \\ {b_{1} = \frac{\left( {g_{0}\left( t_{1} \right) - g_{0}\left( {t_{0} + \lambda } \right) + \theta _{s2,max}} \right) t_{1}}{t_{0} - t_{1}} + g_{0}\left( t_{1} \right) - \theta _{s2,min}} \\ {\lambda = - \frac{\theta _{s2,min}\left( {t_{0} - t_{1}} \right) }{g_{0}\left( t_{1} \right) - g_{0}\left( {t_{0} + \lambda } \right) + \theta _{s2,max}}} \end{matrix} \right. \end{aligned}$$

For the second half of the cycle, the trajectory is constructed by applying symmetry constraints to ensure periodicity. The linear function $k_{2}t+b_{2}$ (red curve in Fig. 9a) is obtained by sequentially reflecting the upstroke function relative to $g_{0}(t)=kt$ and the mid-cycle time $t=\frac{T}{2}$.

For the linear equation $Px+Qy+R=0$ to be axisymmetric with respect to $Dx+Ey+F=0$, there is formula 22.

22 $$\begin{aligned} \frac{Px + Qy + R}{Dx + Ey + F} = \frac{2\left( {PD + QE} \right) }{D^{2} + E^{2}} \end{aligned}$$

For the equation $g_{2}\left( t\right) =k_{1}t+b_{1}$ to be axisymmetric with respect to $g_{0}\left( t\right) =kt$, there is formula 23.

23 $$\begin{aligned} y = \frac{k_{1} - \mu k}{1 - \mu }t + \frac{b_{1}}{1 - \mu },where~\mu = \frac{2\left( {k_{1}k + 1} \right) }{k^{2} + 1} \end{aligned}$$

For axisymmetry about $t=\frac{T}{2}$, there is formula 24.

24 $$\begin{aligned} y = - \frac{k_{1} - \mu k}{1 - \mu }t + \frac{b_{1}}{1 - \mu } + \frac{T}{2}\frac{k_{1} - \mu k}{1 - \mu },where~\mu = \frac{2\left( {k_{1}k + 1} \right) }{k^{2} + 1} \end{aligned}$$

The breakpoints $t_{1}$, $t_{2}$, $t_{3}$ and $t_{4}$ of the motion equation $g_{2}$ are calculated as formula 25 based on the following three principles.

1. $\left[ t_{1},t_{2}\right]$ is the translated linear interval, with the translation amount being $\lambda$.
2. $$g_{2}\left( t_{1}\right) =g_{2}\left( t_{4}\right)$$
3. $$g_{2}\left( t_{2}\right) =g_{2}\left( t_{3}\right)$$
  25 $$\begin{aligned} \left\{ \begin{array}{l} \begin{array}{l} {t_{1} = \frac{T}{4} - \delta + \lambda } \\ {t_{2} = \frac{1}{k_{2}}\left( {k_{1}\left( {\lambda + \frac{T}{4} - \delta } \right) + b_{1} - b_{2}} \right) } \end{array} \\ \begin{array}{l} {t_{3} = \frac{1}{k_{2}}\left( {k_{1}\left( {\lambda - \frac{T}{4} + \delta } \right) + b_{1} - b_{2}} \right) } \\ {t_{4} = \frac{3T}{4} + \delta + \lambda } \end{array} \end{array} \right. \end{aligned}$$
  For the nonlinear part, the parameters are determined by formula 26, and the solution for the nonlinear part of the other half cycle is derived in the same manner.
  26 $$\begin{aligned} \left\{ \begin{matrix} {\omega _{1} = \frac{\pi }{t_{2} - t_{1}}} \\ {A_{1} + B_{1} = 0.5F_{0}} \\ {A_{1}cos\omega _{1}\left( {t_{1} - 0.5\left( {t_{2} + t_{1}} \right) } \right) + B_{1} = k_{1}t_{1} + b_{1}} \end{matrix} \right. \end{aligned}$$
  In summary, the kinematic model for the vein $P_{0}P_{3}$ is fully described by formulas 20, 21, 24, 25, and 26. The solution of the motion equation for the wing vein $P_{0}P_{2}$ is derived analogously. Figure 9b and c demonstrate that the resulting RPA trajectories $(\theta _{s1},\theta _{s2})$ strictly follow the designed monotonic trends during both the flipping (orange regions) and stroke (blue regions) phases, validating the model’s consistency.

### The coordinates of the flapping points in wing-root adjustment mode

**Fig. 10:**
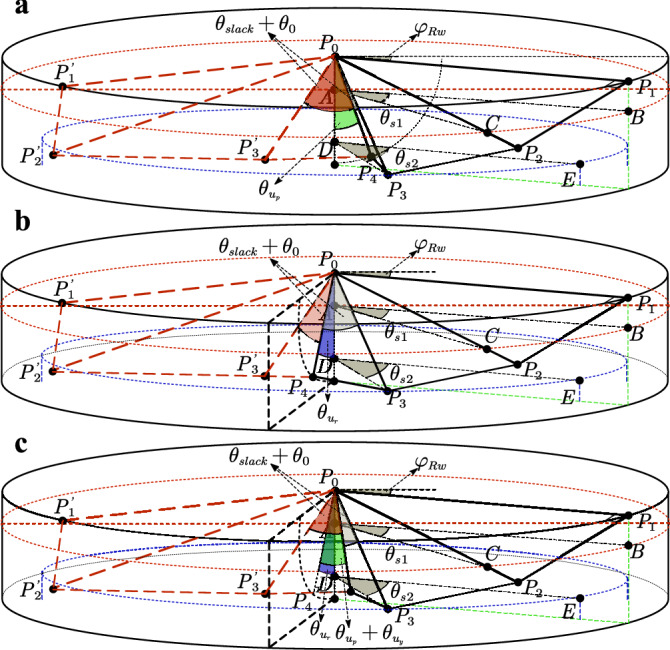
Geometric model of the wing. (**a**) Pitch control mode (**b**) Roll control mode. (**c**) Composite control mode of pitch, roll and yaw.

Next, we present the point coordinate equations. For the pitch control mode, Fig. 10 depicts the geometric models of the wing during flapping under three control modes: pitch control, roll control, and combined pitch-roll-yaw control. In Fig. 10, the three-dimensional wing surface $S_{01^\prime 2^\prime 3^\prime 4}$, shown as a red dashed outline, corresponds to the final flipping process (just before the downstroke). The three-dimensional wing surface $S_{01234}$, drawn with a black solid outline, represents the final downstroke process (just before the flipping).

Formulas 27, 28, and 29 define the point coordinates for $P_{1}$, $P_{2}$, and $P_{3}$ in the pitch control, roll control, and combined pitch-roll-yaw control modes. If the system is in the flipping process, the treatment method from Section 4.2 in equilibrium mode is applied. In this case, the projection displacements of veins $P_{0}P_{2}$ and $P_{0}P_{3}$ along the *z*-axis are neglected. This ensures that, under the given constraint, the kinematic model in the wing-root adjustment mode is mathematically complete.

27 $$\begin{aligned} & P_{1}:\left\{ \begin{matrix} {x_{1} = W_{span}cos\left( \varphi _{Rw} \right) } \\ {y_{1} = W_{span}sin\left( \varphi _{Rw} \right) } \end{matrix} \right. \end{aligned}$$

28 $$\begin{aligned} & P_{2}:\left\{ \begin{matrix} {x_{2}^{2} + y_{2}^{2} + z_{2}^{2} = \left| {P_{0}P_{2}} \right| ^{2}} \\ {\left( {x_{2} - x_{1}} \right) ^{2} + \left( {y_{2} - y_{1}} \right) ^{2} + z_{2}^{2} = \left| {P_{2}P_{1}} \right| ^{2}} \\ {\frac{\left( {x_{2}\vec {i} + y_{2}\vec {j} + 0\vec {k}} \right) \cdot \overrightarrow{P_{0}P_{1}}}{\sqrt{x_{2}^{2} + y_{2}^{2}}\left| {P_{0}P_{1}} \right| } = cos\theta _{s1}} \end{matrix} \right. \end{aligned}$$

29 $$\begin{aligned} & P_{3}:\left\{ \begin{matrix} {x_{3}^{2} + y_{3}^{2} + z_{3}^{2} = \left| {P_{0}P_{3}} \right| ^{2}} \\ {\left( {x_{3} - x_{2}} \right) ^{2} + \left( {y_{3} - y_{2}} \right) ^{2} + \left( {z_{3} - z_{2}} \right) ^{2} = \left| {P_{3}P_{2}} \right| ^{2}} \\ {\frac{\left( {x_{3}\vec {i} + y_{3}\vec {j} + 0\vec {k}} \right) \cdot \overrightarrow{P_{0}P_{1}}}{\sqrt{x_{3}^{2} + y_{3}^{2}}\left| {P_{0}P_{1}} \right| } = cos\theta _{s2}} \end{matrix} \right. \end{aligned}$$

In the composite control mode of pitch, roll and yaw, the calculation formulas for point $P_{4}$ are given by 30, 31, and 32.

30 $$\begin{aligned} & pitch:\left\{ \begin{matrix} {x_{4} = W_{width}sin\left( \theta _{u_{p}} \right) } \\ {z_{4} = W_{width}cos\left( \theta _{u_{p}} \right) } \end{matrix} \right. \end{aligned}$$

31 $$\begin{aligned} & roll:\left\{ \begin{matrix} {y_{4} = W_{width}sin\left( \theta _{u_{r}} \right) } \\ {z_{4} = W_{width}cos\left( \theta _{u_{r}} \right) } \end{matrix} \right. \end{aligned}$$

32 $$\begin{aligned} & pitch \mathrm{ \& } roll\mathrm{ \& } yaw:\left\{ \begin{matrix} {x_{4} = W_{width}sin\left( {\theta _{u_{p}} + \theta _{u_{y}}} \right) } \\ {y_{4} = W_{width}cos\left( {\theta _{u_{p}} + \theta _{u_{y}}} \right) sin\left( \theta _{u_{r}} \right) } \\ {z_{4} = W_{width}cos\left( {\theta _{u_{p}} + \theta _{u_{y}}} \right) cos\left( \theta _{u_{r}} \right) } \end{matrix} \right. \end{aligned}$$

**Fig. 11:**
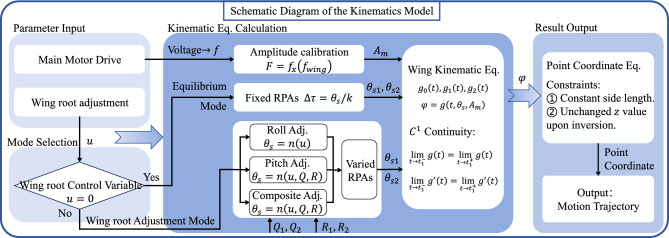
Schematic Diagram of the flapping-wing kinematic model.

Figure 11 presents the schematic diagram of the kinematic model for the FWMAV, which illustrates the complete workflow of kinematic modeling and calculation. The model is divided into four core modules: Parameter Input, Mode Selection, Calculation of Kinematic Equations, and Result Output.

The Parameter Input module consists of two sub-components: the main motor drive input and the wing root adjustment input. Specifically, the main motor drive actuates the periodic flapping of the wing, where different flapping frequencies correspond to distinct flapping amplitudes according to the amplitude calibration equation *F*. Meanwhile, the activation status of the wing root adjustment input determines the operational mode, which is categorized into the equilibrium mode and the wing-root adjustment mode.

In the equilibrium mode, the wing RPAs remain unchanged, allowing for the direct calculation of Kinematic Equation *G*. In contrast, in the wing-root adjustment mode, dynamically varying RPAs are first derived using different RPA equations *N* based on the selected control degrees of freedom, after which the calculation of Kinematic Equation *G* proceeds. Equation *G* outputs the wing flapping angle $\varphi$, followed by the execution of point coordinate calculation and the final generation of the wing motion trajectory.

The above constitutes the complete workflow of the flapping-wing kinematic model proposed in this study.

### A multi-plane aerodynamic simulation method

**Fig. 12:**
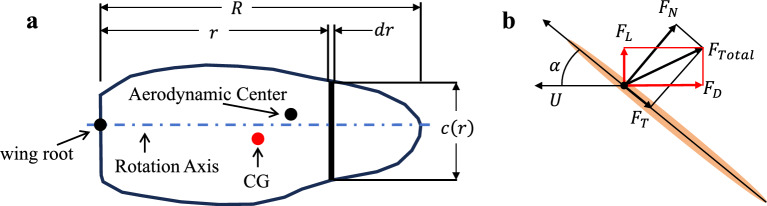
Schematic Diagram of Wing Shape Geometric Parameters. (**a**) Distribution of geometric parameters for a wing of arbitrary shape. (**b**) Force decomposition of the blade element cross-section.

For a rigid planar wing of arbitrary shape, Figure 12 defines the key geometric parameters of the wing. In Fig. 12a, a typical irregular wing shape is shown, while Fig. 12b presents a schematic of the force decomposition acting on a blade element. As shown in Fig. 12a, a chord element is taken at a radial distance *r* from the wing root. Its length, $c\left( r\right)$, can be expressed as a function of *r*, with the width of the element being *dr*, and the wingspan denoted by *R*.

In the model shown in Fig. 12b, *U* represents the velocity vector of the blade element’s cross-section, which is tangent to the blade’s motion trajectory. The angle $\alpha$ between the velocity vector *U* and the blade’s chord line is defined as the aerodynamic attack angle for the blade element. The total aerodynamic force $F_{Total}$ can be decomposed in two coordinate systems: in the normal-tangential coordinate system, it is decomposed into the normal force component $F_{N}$ and the tangential force component $F_{T}$; in the aerodynamic coordinate system, it is decomposed into the lift component $F_{L}$ and the drag component $F_{D}$.

Combining previous studies^19,44,57^, the lift and drag forces induced by the translation motion of a blade element of a two-dimensional rigid plane can be expressed as 33 and 34:

33 $$\begin{aligned} dF_{Ltran}= & \frac{1}{2}\rho C_{L}(\alpha )cr^{2}{\overset{.}{\phi }}^{2}dr \end{aligned}$$

34 $$\begin{aligned} dF_{Dtran}= & \frac{1}{2}\rho C_{D}(\alpha )cr^{2}{\overset{.}{\phi }}^{2}dr \end{aligned}$$

where $\rho$ is the air density, $C_{L}$ is the lift coefficient, $C_{D}$ is the drag coefficient, *c* is the chord length of the blade element, $\phi$ is the flapping angle, and $r\dot{\phi }$ is the cross-sectional velocity of the blade element.

For an arbitrary-shaped wing with a span of *R*, the lift and drag forces of the entire wing can be solved by integrating the lift and drag forces of the blade elements along the span direction. The calculation expressions are shown in formulas 35 and 36:

35 $$\begin{aligned} F_{Ltran}= & {\int {dF_{Ltran}}} = \frac{1}{2}\rho C_{L}(\alpha ){\overset{.}{\phi }}^{2}{\int _{0}^{R}{c(r)r^{2}dr}} \end{aligned}$$

36 $$\begin{aligned} F_{Dtran}= & {\int {dF_{Dtran}}} = \frac{1}{2}\rho C_{D}(\alpha ){\overset{.}{\phi }}^{2}{\int _{0}^{R}{c(r)r^{2}dr}} \end{aligned}$$

The lift and drag coefficients can be obtained from experimental data^57^, and the normal and tangential lift coefficients can be expressed by formula 37:

37 $$\begin{aligned} \begin{matrix} {C_{N}(\alpha ) = 3.4sin\alpha } \\ {C_{T}(\alpha ) = \left\{ \begin{matrix} {0.4{cos}^{2}\left( {2\alpha } \right) } \\ 0 \end{matrix} \right. \begin{matrix} {0 \le \alpha \le \frac{\pi }{4}} \\ {others} \end{matrix}} \end{matrix} \end{aligned}$$

According to the relationship between lift, drag, normal force and tangential force shown in Fig. 12b, the calculation methods of lift coefficient and drag coefficient are shown in formula 38:

38 $$\begin{aligned} \begin{matrix} {C_{L}(\alpha ) = C_{N}(\alpha )cos\alpha - C_{T}(\alpha )sin\alpha } \\ {C_{D}(\alpha ) = C_{N}(\alpha )sin\alpha + C_{T}(\alpha )cos\alpha } \end{matrix} \end{aligned}$$

The following is the aerodynamic effect generated by rotational motion^60^. The circulation of a single blade element due to rotational motion is expressed as formula 39:

39 $$\begin{aligned} dF_{Nrot} = \pi \rho c^{2}\left( {\frac{3}{4} - {\hat{x}}_{0}} \right) r\overset{.}{\phi }\overset{.}{\alpha }dr \end{aligned}$$

The aerodynamic force caused by the rotational motion of the wing is perpendicular to the wing surface, and the rotational circulation needs to be decomposed into the lift and drag directions. For the wing shape involved in this study, the rotation axis coincides with the wing spar, so ${\hat{x}}_{0}$ can be taken as zero, and the expression is shown in formula 40:

40 $$\begin{aligned} \begin{matrix} {dF_{Lrot} = dF_{Nrot}cos\alpha = \frac{3}{4}\pi \rho \overset{.}{\phi }\overset{.}{\alpha }cos\alpha c^{2}(r)rdr} \\ {dF_{Drot} = dF_{Drot}sin\alpha = \frac{3}{4}\pi \rho \overset{.}{\phi }\overset{.}{\alpha }sin\alpha c^{2}(r)rdr} \end{matrix} \end{aligned}$$

Further considering the aerodynamic force caused by the added mass effect, which originates from the reaction force exerted by the surrounding air on the wing during flapping motion. According to the studies by Fung, Sane, Maybury and others^44,61,62^, this force consists of two parts: the force generated by the acceleration of the added mass on the wing surface and the centrifugal force.

The point of application of the surface acceleration force is located at the midpoint of the wing chord, and its mathematical representation is the product of the acceleration at the midpoint of the wing chord and the apparent mass $\rho \pi \frac{c^2}{4}$. The distance from the midpoint of the wing chord to the rotation axis is $\frac{c\left( r\right) }{2}$, and the expression for the surface mass acceleration effect force is shown in formula 41:

41 $$\begin{aligned} dF_{Nacc} = \rho \pi \frac{{c(r)}^{2}}{4}a_{midchord}dr = \rho \pi \frac{{c(r)}^{2}}{4}\left( {\frac{c(r)}{2}\overset{..}{\alpha }\ - r\overset{..}{\phi }sin\alpha } \right) dr \end{aligned}$$

where $a_{midchord}$ represents the acceleration at the midpoint of the wing chord.

The centrifugal force is defined as the product of the apparent mass and $U\dot{\alpha }$, where *U* is the component of the wind speed in the chord direction. Thus, the expression for the surface mass centrifugal force is given by formula 42:

42 $$\begin{aligned} dF_{Ncent} = \rho \pi \frac{{c(r)}^{2}}{4}\left( {- r\overset{.}{\phi }cos\alpha } \right) \overset{.}{\alpha }dr \end{aligned}$$

Orthogonally decomposing the added mass effect along the lift and drag directions gives formula 43:

43 $$\begin{aligned} \begin{matrix} {dF_{Ladd} = \rho \pi \frac{{c(r)}^{2}}{4}\left( {\frac{c(r)}{2}\overset{..}{\alpha }\ - r\overset{..}{\phi }sin\alpha - r\overset{.}{\phi }cos\alpha \overset{.}{\alpha }} \right) cos\alpha dr} \\ {dF_{Dadd} = \rho \pi \frac{{c(r)}^{2}}{4}\left( {\frac{c(r)}{2}\overset{..}{\alpha }\ - r\overset{..}{\phi }sin\alpha - r\overset{.}{\phi }cos\alpha \overset{.}{\alpha }} \right) sin\alpha dr} \end{matrix} \end{aligned}$$

The final solution for the instantaneous lift of a rigid planar wing with an arbitrary shape is the sum of three aerodynamic effects, as shown in formula 44.

44 $$\begin{aligned} \begin{matrix} {F_{L} = {\int _{0}^{R}{dF_{Ltran} +}}dF_{Lrot} + dF_{Ladd}} \\ {F_{D} = {\int _{0}^{R}{dF_{Dtran} +}}dF_{Drot} + dF_{Dadd}} \end{matrix} \end{aligned}$$

During the flapping motion of the wing, the membrane undergoes passive flexible deformation due to the support structures of the wing spar and wing veins. Based on the spatial topological structure of the wing veins, the deformed wing surface can be discretized into several rigid planar elements. It is assumed that the plane $S_{012}$ is formed by the nodes $P_{0}$, $P_{1}$, and $P_{2}$; the plane $S_{023}$ is formed by the nodes $P_{0}$, $P_{2}$, and $P_{3}$; and the plane $S_{034}$ is formed by the nodes $P_{0}$, $P_{3}$, and $P_{4}$, all of which are considered rigid planes. The overall aerodynamic effect of the flexible wing can be approximated by the linear superposition of the aerodynamic effects of these three rigid planes. The aerodynamic effect superposition model is expressed in formula 45.

45 $$\begin{aligned} F_{Total} \approx F_{Total\_ 012} + F_{Total\_ 023} + F_{Total\_ 034} \end{aligned}$$

Existing studies^58,61,62^ show that the added mass effect, while exhibiting a limited enhancement during the flipping process, contributes significantly less than the rotational aerodynamic effects. Throughout the entire flapping cycle, the contribution of the added mass effect is secondary compared to the contributions of the translational and rotational aerodynamic effects. Based on the principle of using a simplified model, this study primarily focuses on analyzing the translational and rotational aerodynamic effects, without performing a quantitative calculation of the added mass effect.

## Simulation

The simulation uses the basic design parameters of the wing as shown in the following table. Table 1 gives the basic design parameters of the wing, where the flapping amplitude is $150^{\circ }$, the nonlinearity parameter $\delta$ is 35 %, and the range of the dimensionless wing-root adjustment quantities (rudder quantities) *u* is $\left[ -1,1\right]$.

**Table 1:** Table 1 Basic design parameters of the wing.

Parameter	Value
Flapping frequence	$[25,35]~Hz$
Wingspan	65 *mm*
Wing width	28 *mm*
Flapping amplitude	$150^{\circ }$
The angle between wing vein 2 and the wing root rotation axis	$35^{\circ }$
Flapping nonlinearity	35 %
Wing-root adjustment quantities $u = \left( {u_{p},u_{r},u_{y}} \right)$	$\left[{- 1,1} \right]$
Slack angle	$13^{\circ }$

### Kinematic simulation

**Fig. 13:**
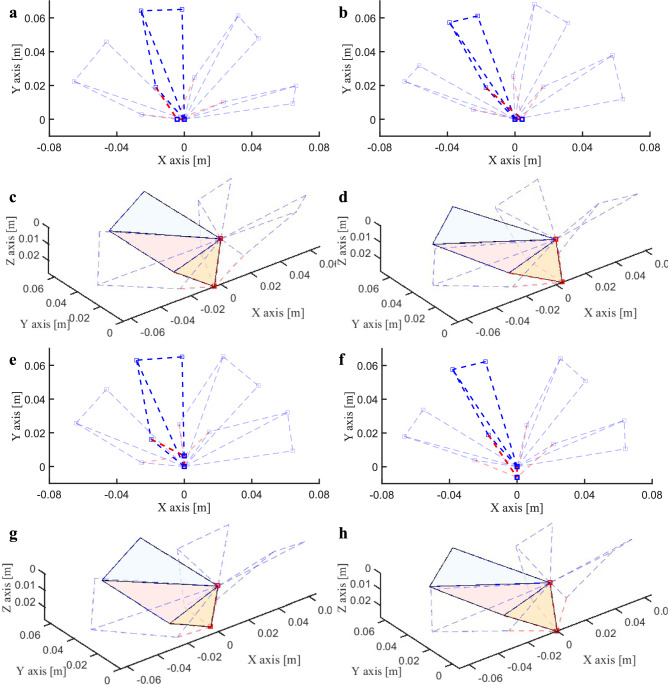
Simulation of wing flapping trajectories. (**a**) Top view of positive full rudder (**b**) Top view of negative full rudder. (**c**) 3D view of positive full rudder (**d**) 3D view of negative full rudder. (**e**) Top view of left full rudder (**f**) Top view of right full rudder. (**g**) 3D view of left full rudder (**h**) 3D view of right full rudder.

The logic of the wing kinematic simulation algorithm under wing-root adjustment is shown in the Supplementary Materials named Algorithm 1 . Next, the simulation results of the wing kinematic model are displayed from the top view and three-dimensional perspective. Figure 13 shows the evolution law of the three-dimensional motion trajectory under the pitch/roll control commands: Figure 13a and c show the positive full rudder output of pitch. During the upstroke process, the wing deformation gradually decreases, and it gradually increases during the downstroke, which leads to uneven deformation of the wing during the up and down strokes, thus generating a pitch control moment. Figure 13b and d show the negative full rudder output of pitch, and the direction of the generated pitch moment is opposite. Figure 13e and g show the left full rudder output of roll, and Fig. 13f and h show the right full rudder output of roll. The deformations of the left and right wings are asymmetric. As the control quantity increases, the deformation of the wing on the rudder side increases, while the deformation of the other wing decreases. The deformation changes of the left and right wings are exactly opposite, thus generating a roll control moment.

### Aerodynamic simulation

**Fig. 14:**
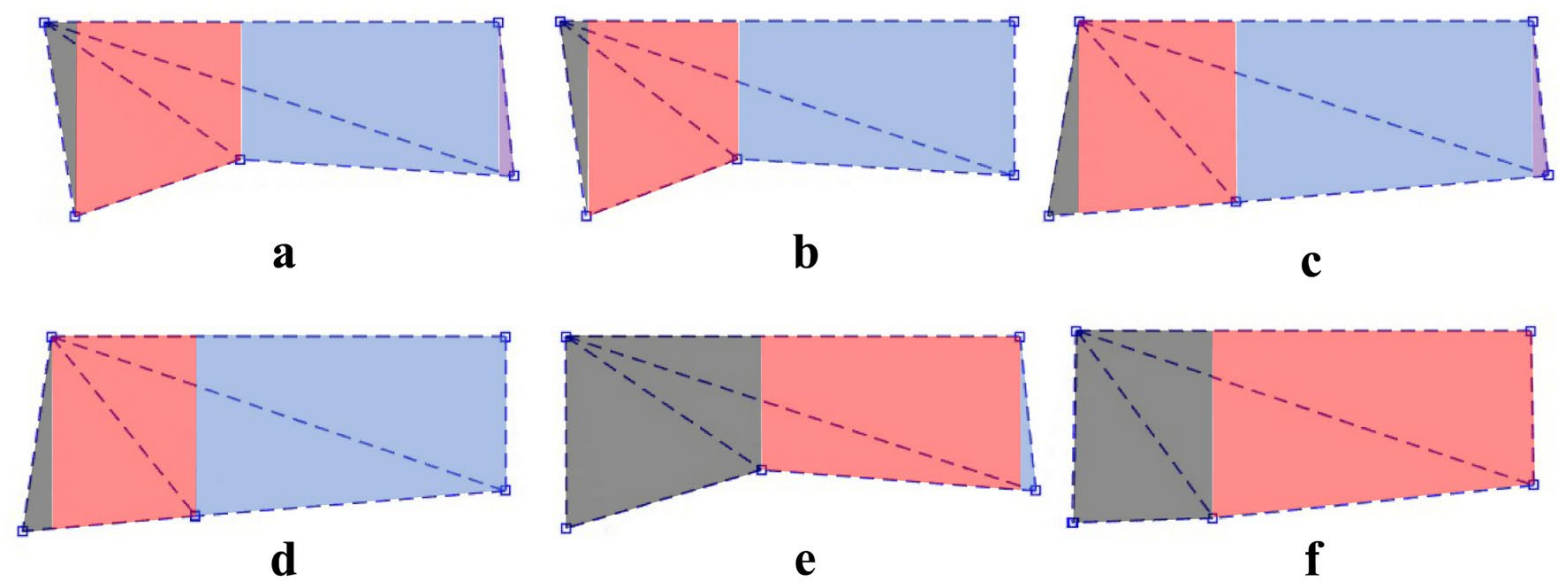
The projections of the wing along the wing coordinate system. (**a**)–(**d**): The projections of the wing under wing-root adjustment mode. (**e**), (**f**): The projections of the wing under equilibrium mode.

Figure 14 shows the projection of the three-dimensional wing surface in the wing coordinate system $Oxyz_{Rw}$. Figure 14a–d correspond to the wing-root adjustment mode, and Fig. 14e, f correspond to the equilibrium mode. Under the assumption of discretized rigid wing surface elements, each projection is decoupled into three rigid planar regions. During the numerical simulation, the wing surface should be partitioned according to the six typical configurations shown in the figure to facilitate the segmented calculation of the aerodynamic effects of blade elements. Taking the projection in Fig. 14a as an example, this morphology is commonly observed in specific flapping phases under the combined pitch-roll-yaw control mode. Using the abscissa of edge points as the boundary, each rigid wing surface element can be divided into four regions. By performing spanwise integration of the aerodynamic effects of blade elements in each region, the numerical solution of the overall aerodynamic characteristics is finally obtained.

**Fig. 15:**
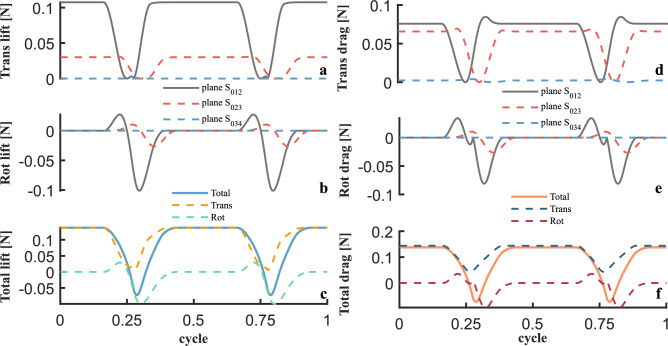
Aerodynamic lift and drag simulation of flexible wing at 35 Hz. (**a**)–(**c**) Translational and rotational instantaneous lift of three planes. (**d**)–(**f**) Translational and rotational instantaneous drag of three planes.

**Fig. 16:**
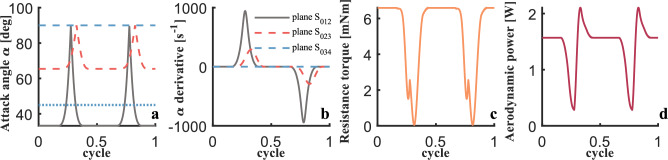
Simulation results of attack angle, drag moment and power of flexible wing at flapping frequency of 35 Hz. (**a**) Instantaneous attack angle. (**b**) Derivative of instantaneous attack angle. (**c**) Instantaneous drag moment. (**d**) Instantaneous aerodynamic power.

The corresponding algorithm flow is shown in the in the Supplementary Materials named Algorithm 2. Next, the aerodynamic simulation results of the flexible wing under the assumption of discretized rigid wing surface elements are shown in Figs. 15 and 16. Among them, Figure 15a–c show the instantaneous lift of the wing, and the peak value of the combined lift from the translational and rotational parts can reach 14 *g*, as shown in Fig. 15c. Figure 16a, b show the curves of the attack angle and the derivative of the attack angle for the rigid planar elements, with the blue dotted line being the $45^{\circ }$ critical attack angle. According to the variation law of the lift and drag coefficient^57^, when the attack angle exceeds the critical value of $45^{\circ }$, the lift coefficient shows a decreasing trend while the drag coefficient continues to increase. The plane $S_{012}$ maintains an angle of attack of approximately $18^{\circ }$, and the attack angle can reach $90^{\circ }$ during the flipping process, while the attack angle of the plane $S_{023}$ remains above $45^{\circ }$ continuously. By comparing the aerodynamic contributions of the planes $S_{012}$, $S_{023}$, and $S_{034}$ with the variation trend of the attack angle in Fig. 15a, b, and a, it can be seen that the main regions providing lift are $S_{012}$ and $S_{023}$, while the attack angle of $S_{034}$ remains at $90^{\circ }$ for a long time, basically making no aerodynamic contribution.

Figure 15d–f show the instantaneous aerodynamic drag characteristics of the wing. As shown in Fig. 15d, the contribution of $S_{023}$ to the drag is not low, also because the attack angle of $S_{023}$ remains above $45^{\circ }$ for a long time. Similarly, since $S_{034}$ is relatively close to the wing root, the linear velocity is small, and the aerodynamic drag generated is basically negligible.

As shown in Figure 16c, the peak value of the instantaneous drag moment of the wing is estimated to reach 7 $mN \cdot m$, which provides a key mechanical criterion for the material selection of the transmission rope of the rope-driven structure. Figure 16d shows that the average power consumption of a single wing is 1.4689 *W*, and the power reaches a peak of about 2.2 *W* during the flipping process.

**Fig. 17:**
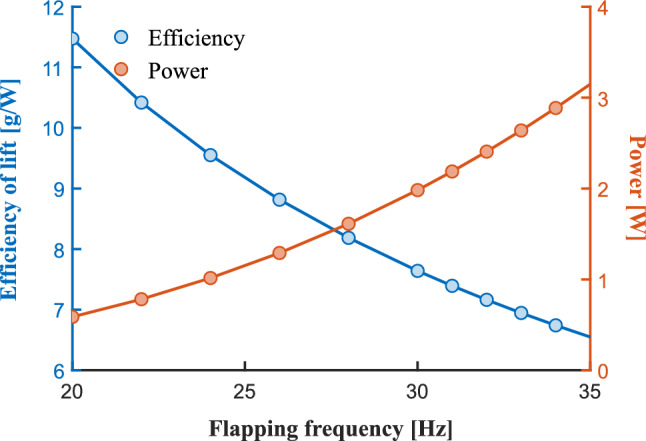
Prediction of wing aerodynamic efficiency by multi-rigid plane method.

Figure 17 shows the prediction curves of wing aerodynamic efficiency and power consumption by the multi-rigid plane method. Here, the wing aerodynamic efficiency $\eta$ is defined as the ratio of the average lift $\bar{L}$ to the average power consumption $\bar{P_{w}}$, as shown in formula 46, with the unit of *g*/*W*. The results show that the power consumption increases monotonically with the increase of flapping frequency, while the aerodynamic efficiency decreases monotonically. Through this prediction curve, the optimal balance point between force efficiency and power can be determined in the wing design stage, realizing the optimization of aerodynamic efficiency under the premise of meeting the lift requirement, which provides strong support for the wing aerodynamic design.

46 $$\begin{aligned} \eta = \frac{\overset{-}{L}}{\overset{-}{P_{w}}} \end{aligned}$$

**Fig. 18:**
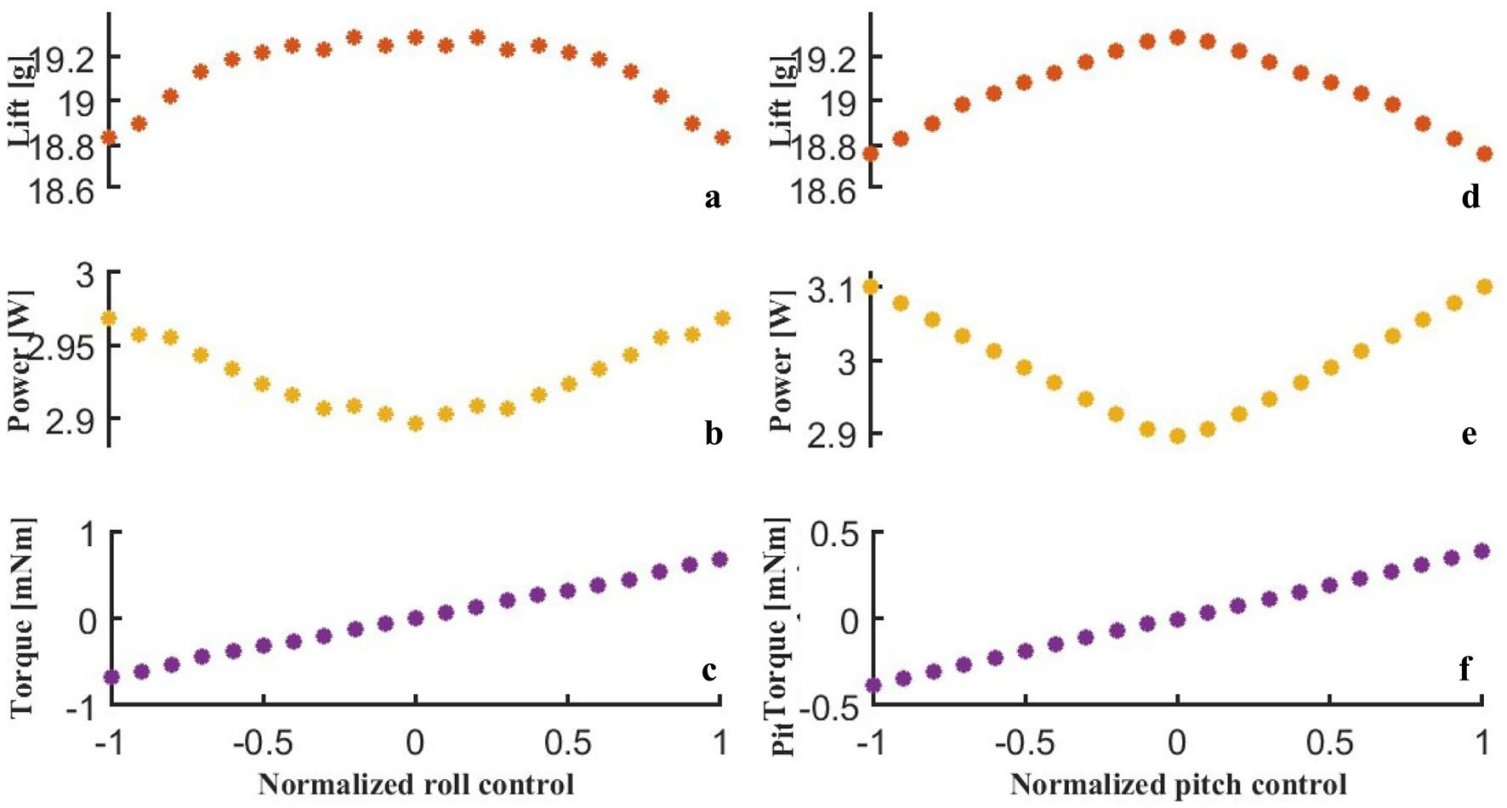
Aerodynamic moment responses under wing-root adjustment mode at 35 Hz. (**a**)–(**c**) Lift, power, and roll moment responses under roll rudder output. (**d**)–(**f**) Lift, power, and pitch moment responses under pitch rudder output.

The following presents the aerodynamic moment response under the wing-root adjustment mode at a flapping frequency of 35 *Hz*. Figure 18a–c and d–f show the results of roll and pitch wing-root adjustment, respectively. Figure 18a is the total lift curve under roll rudder output. When the roll rudder is in the interval of $[- 0.5,0.5]$, the total lift is basically maintained at about 19.2 *g*. As the rudder output continues to increase, the lift shows a downward trend. For pitch control, with the increase of the pitch rudder, the lift also decreases slightly, as shown in Fig. 18d. In the subsequent controller design, the influence of the rudder output on the lift should be considered, and the lift should be compensated accordingly.

Figure 18b and e reflect the curves of average power consumption varying with the rudder output. The larger the command, the greater the power consumption. Figure 18c shows the roll moment curve, and it can be considered that the roll control moment has a linear relationship with the roll command $\left( R^{2}\ge 0.9993\right)$. Figure 18f shows the pitch moment curve, and the simulated moment also has a linear relationship with the command $\left( R^{2}\ge 0.9999\right)$.

## Experiment and results

**Fig. 19:**
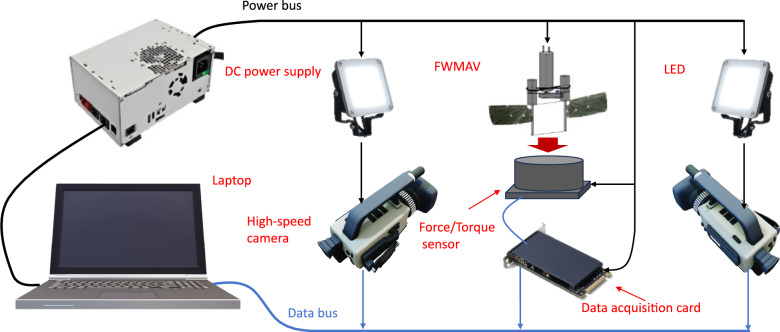
Dual-camera high-speed photography measurement system.

Experimental Setup 1: The architecture of the dual-camera high-speed photography measurement system is shown in Fig. 19, with the specific configuration as follows:

Two ACS-3 M16E high-speed cameras: Resolution of 1280$\times$720 *pixels*, frame rate of 5000 *fps*.

Two sets of high-brightness LED area light sources.

Data acquisition workstation: The system ensures no less than 150 *fps* effective images within a single flapping cycle at the maximum flapping frequency of 35 *Hz*, and achieves frame-loss-free acquisition with a 32 *GB* high-speed cache.

Six-dimensional force sensor and acquisition card: Using a Nano17 six-axis force sensor with a force measurement accuracy of 0.147 *g* and a moment accuracy of 0.0069 $mN \cdot m$.

Image processing employs the TEMA motion analysis system, realizing 3D marker point trajectory reconstruction through a centroid tracking algorithm and correlation matching algorithm. The system can output time-series data of displacement, velocity, acceleration, and angle parameters, and supports multimodal data visualization.

**Fig. 20:**
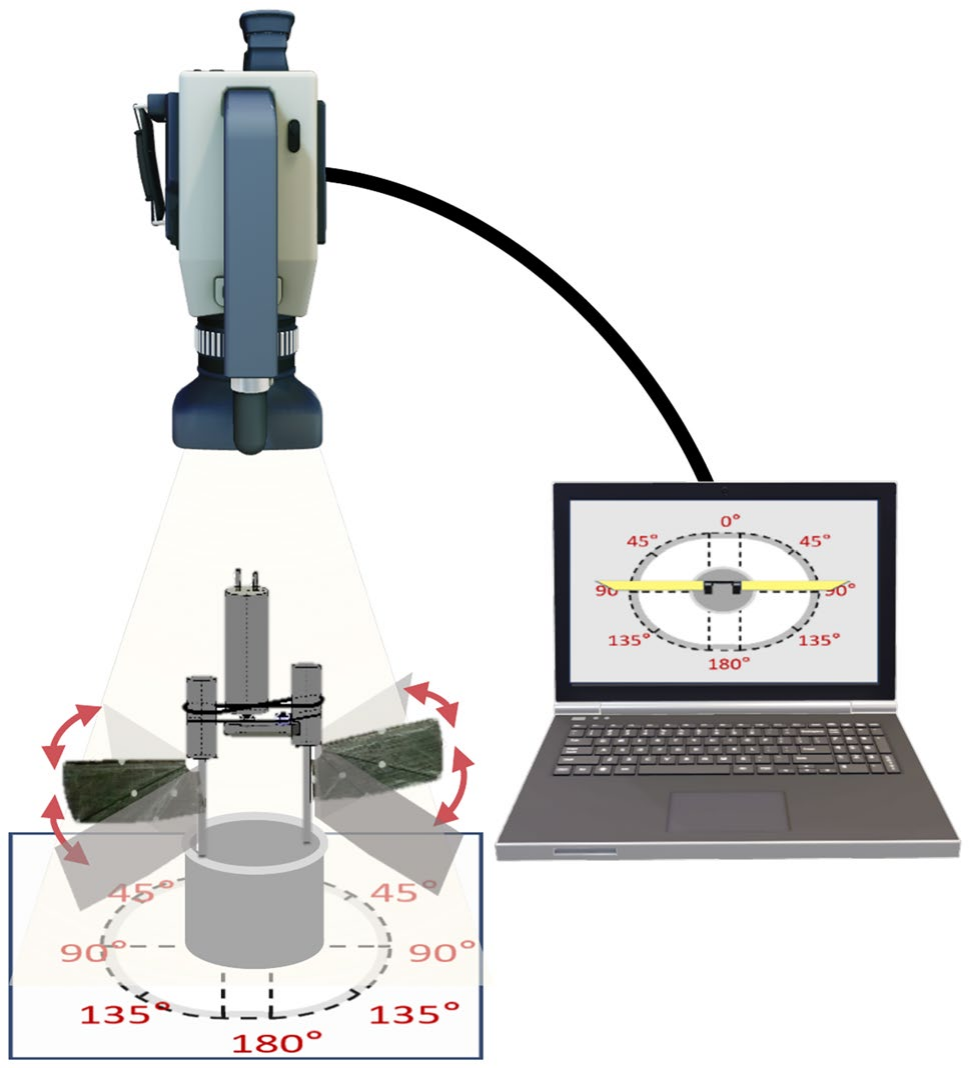
Single-camera vertical photography measurement system.

Experimental Setup 2: The architecture of the single-camera vertical photography measurement system is shown in Fig. 20, with the specific configuration as follows:

One ACS-3 M16E high-speed camera. Two sets of high-brightness LED area light sources. Data acquisition workstation. Six-dimensional force sensor and acquisition card. Angle dial for directly reading the flapping amplitude and relaxation phase angle.

The system is composed of a high-speed photography unit and an angle calibration plate. The photography unit is vertically mounted directly above the flapping wing mechanism, and the optical axis is ensured to be orthogonal to the wing flapping plane through dual-camera calibration. This configuration enables synchronous measurement of the wingtip flapping trajectory and the chordwise deformation of the flexible wing.

### Wing flapping experiment in equilibrium mode

**Fig. 21:**
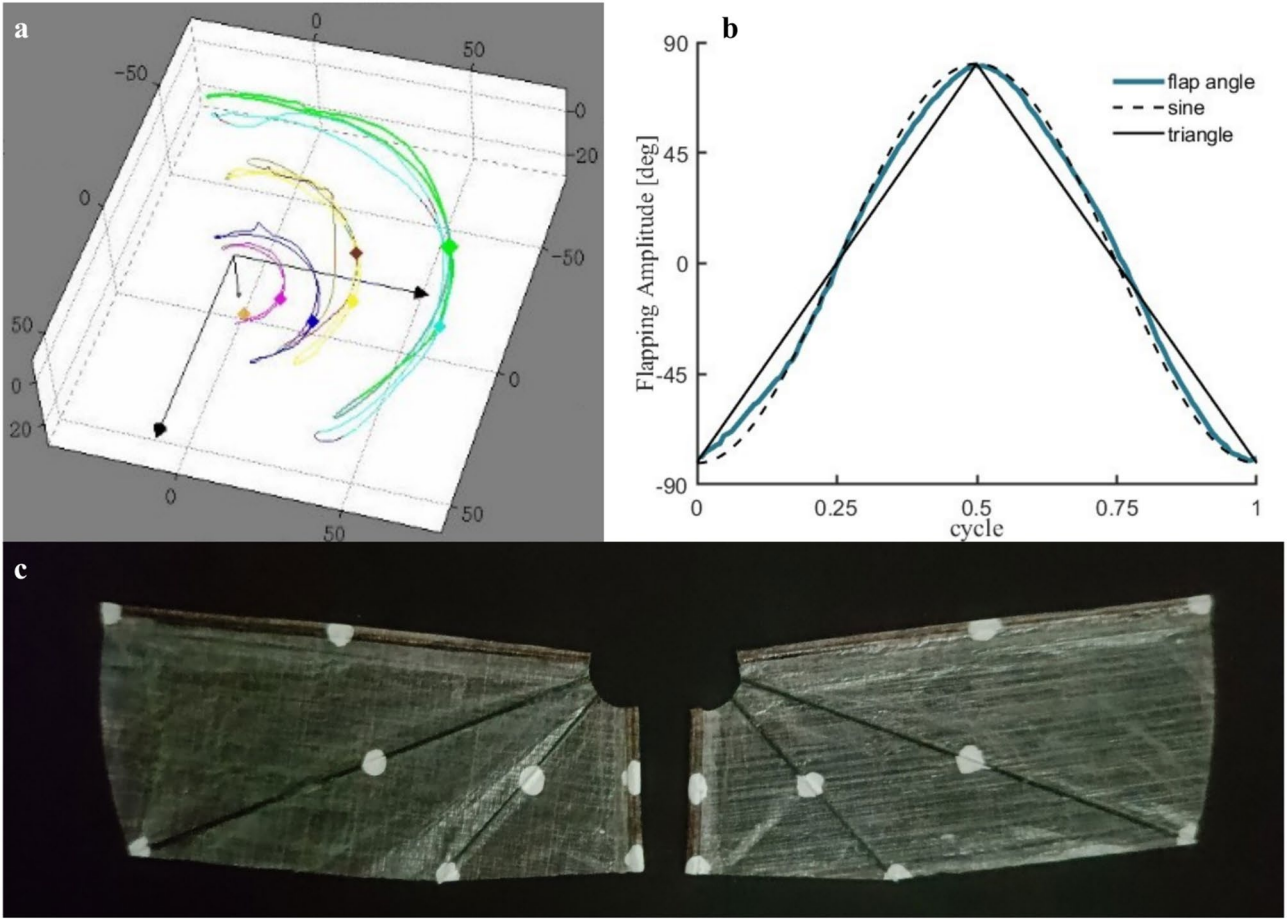
Wing Flapping Experiment in Balanced Mode. (**a**) Actual flapping trajectory of the wing. (**b**) Measured flapping angle curve of the wing outer edge point. (**c**) Wing with reflective markers.

In equilibrium mode, the wing root remains fixed without torsion, and the wing spar and veins undergo periodic flapping around the wing root axis $P_{0}P_{4}$. Experiment Setup 1 was used for this experiment. Figure 21a shows the actual flapping trajectory captured by the high-speed photography system. Figure 21b presents the flapping angle curve reconstructed based on the trajectory of the outer edge point of the wing spar when the motor is driven by a constant voltage. The solid line in the figure represents the triangular wave signal, and the dashed line represents the sine wave signal. The flapping trajectory is basically within the envelope of these two signals. To accurately quantify the wing deformation, reflective markers were placed at key positions on the wing spar and veins, as shown in Fig. 21c.

Based on the experimental data shown in Fig. 21b, the flapping frequency is 35 *Hz*, and the flapping period *T*=0.0286*s*. The kinematic equation *G* of the wing, that is, formulas 4 and 5, is used for fitting, and the fitting result is shown in formula 47 as follows.

To evaluate the goodness of fit of the kinematic equations to the experimental trajectory data, we compare the coefficients of determination ($R^2$) and normalized root-mean-square errors (NRMSE) between the method proposed in this paper, Berman’s method^48^, and the experimental data. Specifically, the $R^2$ and NRMSE of the proposed method are 0.9888 and 3.58%, respectively, while those of Berman’s method are 0.9954 and 2.31%. A comparison of these metrics shows that the accuracy of the kinematic equations proposed in this paper is slightly lower than that of Berman’s method, but the discrepancy is fully acceptable. This statistical evidence confirms that the proposed equations can accurately describe the wing motion.

47 $$\begin{aligned} g_{0}(t) = \left\{ {\begin{array}{l} \begin{array}{l} {14035t} \\ {22.34cos\left( {628.3\left( {t - 0.0071} \right) } \right) + 65.16} \end{array} \\ \begin{array}{l} {- 14035\left( {t - 0.0143} \right) } \\ {22.34cos\left( {628.3\left( {t - 0.0264} \right) } \right) - 65.16} \\ {14035\left( {t - 0.0286} \right) } \end{array} \end{array}~~~~~\begin{array}{l} \begin{array}{l} {0 \le t< 0.0046} \\ {0.0046 \le t< 0.0096} \end{array} \\ \begin{array}{l} {0.0096 \le t< 0.0189} \\ {0.0189 \le t< 0.0239} \\ {0.0239 \le t < 0.0286} \end{array} \end{array}} \right. \end{aligned}$$

### Amplitude calibration experiment

**Fig. 22:**
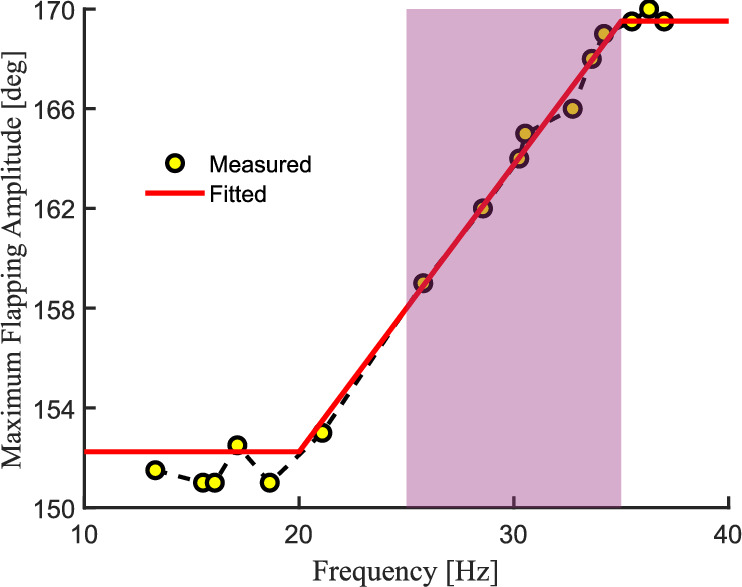
Amplitude-Frequency Characteristic Curve (Test Frequency Range: 10–40 *Hz*).

The purpose of the amplitude calibration experiment is to account for the influence of elastic deformation of the drive structure’s transmission rope on the flapping amplitude. Using Experimental Setup 2, Fig. 22 shows the calibration curve of the wing flapping amplitude varying with frequency. The experimental results indicate that: in the low-frequency region $\left( f<20Hz\right)$ and the high-frequency region $\left( f>35Hz\right)$, the amplitude stabilizes at $F_0\ \pm \ 0.8\%$; in the transition region $\left( f\in \left[ 20,35\right] Hz\right)$, the amplitude approximately increases linearly with frequency, and its dynamic characteristics can be accurately characterized by the linear regression model shown in formula 48.

48 $$\begin{aligned} F_{0} = 1.1514*f + 129.2134~~~~~~~~~f \in [20,35]\end{aligned}$$

### $\textbf{z}$-Axis projection experiment of wing spar and veins

**Fig. 23:**
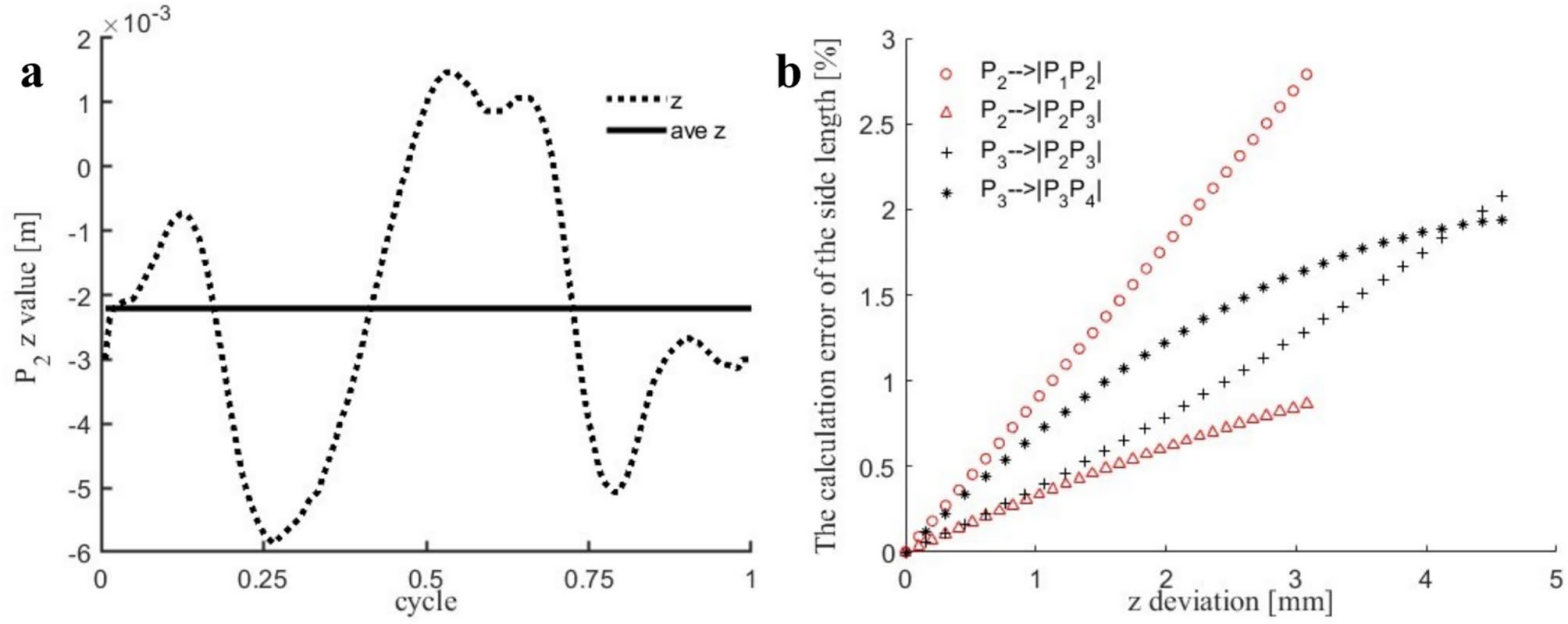
*z*-Axis Projection Experiment of Wing Spar and Veins. (**a**) *z*-axis projection curve of wing vein point $P_{2}$. (**b**) Influence of *z*-axis fluctuations of $P_{2}$ and $P_{3}$ on the solution of side lengths of planes $S_{012}$, $S_{023}$, and $S_{034}$ (Horizontal axis: *z*-axis displacement $\left[ mm\right]$; Vertical axis: relative error of side length estimation).

During the actual flapping process, the *z*-axis projection of the wing spar and veins exhibits a characteristic flattened “8”-shaped trajectory (see Supplementary Video 1). To determine the influence of *z*-axis fluctuations of the wing spar and veins on the solution of the three-plane side lengths, a *z*-axis projection experiment for the wing spar and veins was conducted using Experimental Setup 1. As shown in Fig. 23a, the fluctuation amplitude of point $P_{2}$ in the *z*-axis projection of the wing vein $P_{0}P_{2}$ is less than 3.5 *mm*, with an average fluctuation error of 1.9 *mm*. Here, we present the influence of the upward and downward *z*-axis fluctuations of $P_{2}$ and $P_{3}$ on the solution of the side lengths of planes $S_{012}$, $S_{023}$, and $S_{034}$. Figure 23b shows that when the calculation relative errors of the side lengths of planes $S_{012}$, $S_{023}$, and $S_{034}$ caused by the *z*-axis fluctuations of points $P_{2}$ and $P_{3}$ are less than 1.8 %, i.e., $\mathrm {\Delta z}\ \le \ 2.0\ mm$. Based on the above analysis, the constraint condition is established: the projection displacements of the wing veins $P_{0}P_{2}$ and $P_{0}P_{3}$ on the *z*-axis are negligible. The kinematic model under this constraint condition has mathematical completeness.

### The relaxation phase angle measurement experiment

**Fig. 24:**
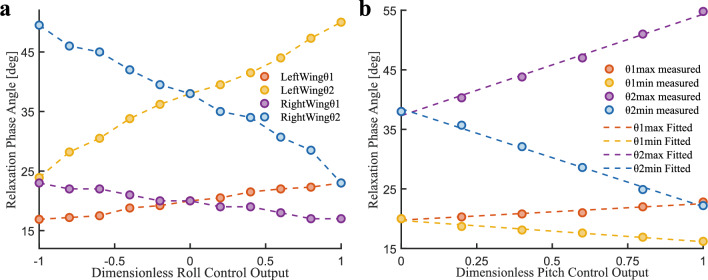
Curves of RPAs in roll and pitch control modes. (**a**) Variation of $\theta _{s1}$ and $\theta _{s2}$ with normalized roll rudder output $u_{r}$. (**b**) Variation ranges of $\theta _{s1}$ and $\theta _{s2}$ with normalized pitch rudder output $u_{p}$.

Using experimental setup 2, the phase difference between the wing spar and wing veins, namely the relaxation phase angle (RPA) data, was obtained based on the top-down perspective of a high-speed camera. Figure 24 below shows the variation law of the RPAs under the roll and pitch wing-root adjustment modes.

For the roll control mode, as the rudder is output to one side, the wing deformation on one side gradually decreases, and the corresponding one on the other side increases. Figure 24a shows the curve of the RPAs between the wing spar and wing veins changing with the control rudder output in roll control mode. When the control rudder is zero (i.e., $u_{r}$=0), the RPA $\theta _{s1}$ between $P_{0}P_{2}$ and $P_{0}P_{1}$ is $20^\circ$, and the RPA $\theta _{s2}$ between $P_{0}P_{3}$ and $P_{0}P_{1}$ is $38^\circ$. With the increase of the control rudder, the RPA of the left wing increases, while that of the right wing decreases, showing a basically linear change trend. The linearity of $\theta _{s1}$ is $R^{2}$=0.9892, and that of $\theta _{s2}$ is $R^{2}$=0.9894. Through linear fitting, the RPA equations of the two wings under the roll control mode are shown in formula 49. By comparing the left and right wings, due to the inevitable asymmetric installation of the structure, the fitted RPA equations of the left and right wings are not completely consistent.

49 $$\begin{aligned} \begin{matrix} {Left~wing\left\{ \begin{matrix} {\theta _{s1} = 3.2318\textbf{u}_{\textbf{r}} + 19.9} \\ {\theta _{s2} = 12.0955\textbf{u}_{\textbf{r}} + 37.5364} \end{matrix} \right. } \\ {Right~wing\left\{ \begin{matrix} {\theta _{s1} = - 3.0455\textbf{u}_{\textbf{r}} + 19.8182} \\ {\theta _{s2} = - 12.0864\textbf{u}_{\textbf{r}} + 37.3818} \end{matrix} \right. } \end{matrix} \end{aligned}$$

The pitch control mode is shown in Fig. 24b. As the control rudder $u_{p}$ increases, both the maximum RPAs $\left( \theta _{s1,max},\theta _{s2,max}\right)$ and the minimum RPAs $\left( \theta _{s1,min},\theta _{s2,min}\right)$ within a cycle exhibit approximately linear growth or decline. The established model is as shown in formula 50.

50 $$\begin{aligned} \left\{ \begin{array}{l} \begin{array}{l} {\theta _{s1,max} = 2.7571\textbf{u}_{\textbf{p}} + 19.7714} \\ {\theta _{s1,min} = - 3.5571\textbf{u}_{\textbf{p}} + 19.6952} \end{array} \\ \begin{array}{l} {\theta _{s2,max} = 17.0429\textbf{u}_{\textbf{p}} + 37.2952} \\ {\theta _{s2,min} = - 16.4143\textbf{u}_{\textbf{p}} + 38.4571} \end{array} \end{array} \right. \end{aligned}$$

Combined with formulas 16, 17 and 50, the RPA equation *N* under the pitch control mode is shown in formula 51:

51 $$\begin{aligned} \left\{ \begin{matrix} {\theta _{1} = \frac{6.3142\textbf{u}_{\textbf{p}} + 0.0762}{\varphi _{Rwv_{1},t_{q1}} - \varphi _{Rwv_{1},t_{q2}}}\mathbf {\varphi }_{\textbf{R}\textbf{w}\textbf{v}_{1}} - \frac{\theta _{s1,max}\varphi _{Rwv_{1},t_{q2}} - \theta _{s1,min}\varphi _{Rwv_{1},t_{q1}}}{\varphi _{Rwv_{1},t_{q1}} - \varphi _{Rwv_{1},t_{q2}}}} \\ {\theta _{2} = \frac{33.4572\textbf{u}_{\textbf{p}} - 1.1619}{\varphi _{Rwv_{2},t_{r1}} - \varphi _{Rwv_{2},t_{r2}}}\mathbf {\varphi }_{\textbf{R}\textbf{w}\textbf{v}_{2}} - \frac{\theta _{s2,max}\varphi _{Rwv_{2},t_{r2}} - \theta _{s2,min}\varphi _{Rwv_{2},t_{r1}}}{\varphi _{Rwv_{2},t_{r1}} - \varphi _{Rwv_{2},t_{r2}}}} \end{matrix} \right. \end{aligned}$$

### Aerodynamic experiment in wing-root adjustment mode

**Fig. 25:**
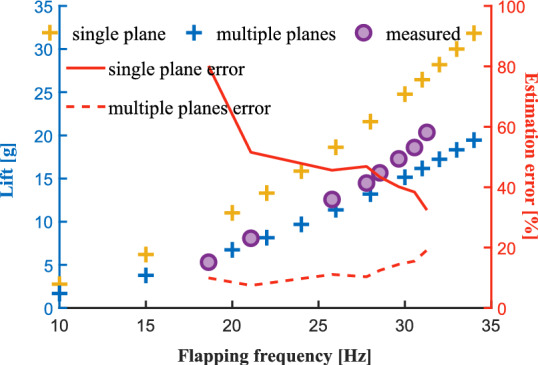
Comparison between lift predictions of rigid plane and multi-rigid plane and measured lift. The left vertical axis shows the lift comparison, and the right vertical axis shows the estimation errors of the two methods.

To verify the aerodynamic model of the wing in wing-root adjustment mode, experimental setup 2 was used. Figure 25 shows the comparison between the predicted lift and the measured lift. The left vertical axis is the lift comparison, in which the purple discrete points represent the lift curve measured in the experiment, and the yellow curve and blue curve represent the single-plane method and the multi-plane method. The right vertical axis is the estimation errors of the two methods. The red solid line is the single-plane estimation error, and the red dashed line is the multi-plane estimation error. The prediction accuracy of the multi-plane method is better than that of the single-plane method, and the error does not exceed 20 % in the range of 20-30 *Hz*. This is mainly because the single-plane method only uses one angle of attack to describe the windward angle of the plane, and we use a fixed angle $45^\circ$ for calculation, so the single-plane prediction result is much larger than the measured lift. The multi-plane method can use multiple time-varying angles of attack to approximate the windward angle of the flexible wing, thus making the prediction closer to the reality.

**Fig. 26:**
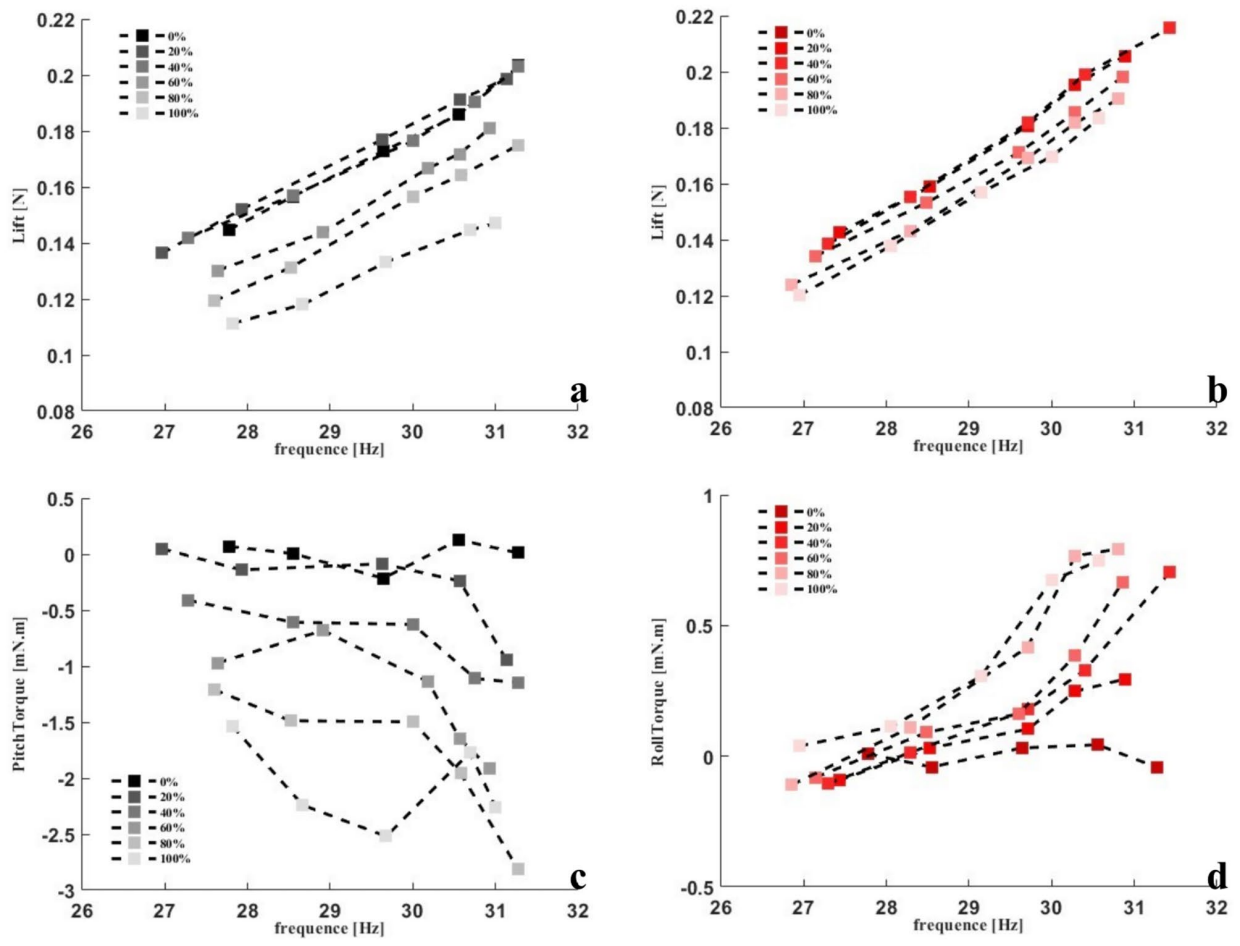
Experimental measurement results. (**a**), (**b**) Lift variation curves under the influence of pitch and roll wing-root adjustments and flapping frequency. (**c**), (**d**) Moment variation curves under the influence of pitch and roll wing-root adjustments and flapping frequency.

Figure 26a, b present the lift variation curves under the influence of pitch and roll wing-root adjustments as well as flapping frequency, where each curve shows the effect of different rudder outputs. When the pitch and roll wing-root adjustment rudders increase from 0 to 100 %, the lift shows a decaying trend under the same flapping frequency. The lift decay amplitude caused by pitch wing-root adjustment is higher than that by roll adjustment, with the maximum drop reaching 5 *g*. This phenomenon is in good agreement with the trend predicted by the model, as shown in Figure 18a and d. Figure 26c–d present the moment variation curves under the influence of pitch and roll wing-root adjustments as well as flapping frequency: the moment shows a monotonically increasing trend with the increase of flapping frequency. Under the fixed flapping frequency, the increase of adjustment rudder can effectively improve the moment output, and this characteristic is reproduced in the simulation, as shown in Fig. 18c and f. The current model prediction accuracy still needs further optimization, and relevant improvements will be taken as the focus of follow-up research.

###  Error analysis and future work

Although the proposed model demonstrates a good agreement with the experimental data, a discrepancy of up to 20% in lift estimation is observed. This error can be attributed to three main aspects: measurement limitations, kinematic modeling simplifications, and aerodynamic assumptions.

First, regarding measurement, the ATI Nano17 sensor used in the experiments has a z-axis resolution of 0.147 *g*. While multi-cycle averaging was employed to mitigate random noise, the inherent sensor precision introduces a measurement uncertainty of approximately 0.5% relative to the baseline hovering lift.

Second, the limitations of the kinematic model constitute a primary source of error. The current model relies on a linear mapping between the flapping frequency and amplitude to account for the rope elasticity in the drive mechanism. This linear simplification does not fully capture the non-linear dynamic response of the flexible structure. Furthermore, the core innovation of this study involves approximating the continuous deformation of the flexible wing using a segmented function based solely on the spar and vein trajectories. This method effectively balances computational efficiency with modeling complexity, making it highly suitable for the proposed wing-root adjustment mechanism. However, this trade-off results in a loss of spatial resolution, as the complex passive deformation of the wing surface is represented by a limited number of characteristic lines rather than a full surface reconstruction.

Third, the aerodynamic simulation employs a multi-plane method based on Blade Element Theory (BET). By treating the flexible wing as a combination of rigid planes, this approach achieves high computational speed but yields lower accuracy compared to Computational Fluid Dynamics with Fluid-Structure Interaction (CFD-FSI).

To address these limitations and further reduce the estimation error, future work will focus on the following modifications:

1. Hardware Upgrade: Utilizing higher-precision sensors (e.g., Nano17Ti) with optimized ranges to minimize data acquisition quantization errors.
2. Model Refinement: Improving the kinematic accuracy by replacing the linear amplitude calibration with high-order polynomial fitting. Additionally, the kinematic model can be enhanced by increasing the number of “virtual veins” to divide the wing into finer sectors or by adopting a conical surface approximation. This would provide a more precise description of the flexible wing’s passive deformation, reducing geometric errors.
3. Aerodynamic Optimization: The application of the Unsteady Panel Method (UPM) is explored to further balance computational efficiency and accuracy in aerodynamic force calculation.

## Conclusion

In response to the described gaps in three-dimensional kinematic modeling of flexible wings under wing-root adjustment and the difficulty of quantifying their aerodynamic effects, this paper makes the following contributions:

1. Kinematic trajectory function for wing spar and veins. We introduce a novel motion equation that describes the coupled movement of the wing spar and wing veins. This equation combines triangular-wave and sinusoidal-wave parameters into a single trajectory function, and uses the phase difference between spar and vein projections to quantify three-dimensional flexible deformation of the wing.
2. Quantitative representation of wing-root adjustment. By analyzing how wing-root adjustment alters wing flexible deformation, we derive a quantitative mapping between the magnitude of root adjustment and the resulting degree of flexible deformation across the wing surface.
3. A rigid division method for flexible wing via the wing vein topology. To enable efficient aerodynamic simulation, we partition the flexible wing into rigid, planar elements guided by the spatial topology of the vein network. Under quasi-steady assumptions, each dynamically deforming surface is discretized into two-dimensional rigid panels, allowing blade-element-style aerodynamic load calculations.
4. Aerodynamic simulation of wing-root adjustment flapping. Using the wing-root-adjusted kinematic model as input, we perform aerodynamic simulations and observe that, under a fixed flapping frequency, increased root adjustment causes a slight decrease in lift while producing a control moment that scales linearly with the adjustment magnitude.
5. Experimental validation. We conduct flapping experiments with controlled pitch and roll adjustment of the wing root to measure lift and aerodynamic moments across a range of flapping frequencies. The experimental trends agree closely with model predictions: lift estimation errors remain below 20 %, and our multi-plane method improves lift-prediction accuracy by at least 20 % compared to single-plane blade-element models.

Together, these advances significantly enhance the predictive accuracy of flexible-wing aerodynamics under wing-root adjustment, extend the research boundaries in three-dimensional kinematic and aerodynamic modeling of flexible wings under the wing-root adjustment, and provide a practical computational framework for rapid aerodynamic design and controller development.

## Supplementary Information

Supplementary Information.

## Data Availability

All data generated or analysed during this study are included in this published article [and its supplementary information files].
