# Supplementary material for: Kinematic and aerodynamic modeling of flexible wings with wing root adjustment for flapping wing micro aerial vehicles
Source: Sci Rep. 2026 Mar 2;16:9827. doi: 10.1038/s41598-026-40582-8 (PMC13018484; doi:10.1038/s41598-026-40582-8)
Supplement: Supplementary file 1 — Supplementary Information. [file 41598_2026_40582_MOESM1_ESM.zip › supplementary/Algorithm 1.docx]

Algorithm 1: Wing Flapping Trajectory Generation Calculation

This algorithm is responsible for generating the wing movement trajectory. Its input parameters include wing geometric parameters, flapping frequency, and the equation root solving search domain $\Delta_{eqn}$ (used to control the convergence accuracy of equation root solving), and finally outputs the wing point movement trajectory.

| Algorithm 1: Generation of wing kinematics |
| --- |
| Input:$f$,$\theta_{slack}$,$u$,$W_{span}$,$W_{width}$,$\Delta_{eqn}$,$\theta_{plane1}$,$\theta_{plane2}$  Output: trajectory of $P_{0}-P_{4}$  1 Calculation of biomechanical wing parameters  2\| $\theta_{p}⟵\theta_{plane1}+\theta_{plane2}$  3\| $P_{0}P_{1}⟵W_{span}$  4\| $P_{1}P_{2}⟵W_{width}$  5\| $P_{0}P_{2}⟵sqrt\left( P_{0}P_{1}^{2}+P_{1}P_{1}^{2} \right)$  6\| $P_{0}P_{3}⟵W_{width}/\left( \mathrm{co}s \left( \theta_{p} \right) \right)$  7\| $P_{2}P_{3}⟵W_{span}-P_{0}P_{3}*sin\left( \theta_{p} \right)$  8\| $P_{0}P_{5}⟵W_{width}$  9\| $P_{3}P_{5}⟵sqrt\left( P_{0}P_{3}^{2}+P_{0}P_{5}^{2}-2*P_{0}P_{3}*P_{0}P_{5}*cos\left( \theta_{slack}+\theta_{p} \right) \right)$  10 $F⟵Amplitude_{c}alibration\left( f \right)$  11 $\left[ \theta_{s1},\theta_{s2} \right]⟵RPA_{c}alculate\left( u \right)$  12 $\left[ \varphi_{0},\varphi_{1},\varphi_{2} \right]⟵\varphi_{c}alculate\left( F,f,\theta_{s1},\theta_{s2},u \right)$  13 $P_{t}rajectory⟵P_{c}alculate\left( \varphi,Geometric parameters of the wings \right)$  14 while(flap_state goes through a complete flapping cycle)  15 \| if flap_state = downstroke or upstroke process  16 \| \| $P^{sp}⟵vpasolve\left( eqns,P_{i}^{sp},P_{i-1}^{sp}\pm\Delta_{eqn} \right)$  17 \| else if flap_state = flipping process  18 \| \| $P_{i,z}^{sp}⟵P_{i-1,z}^{sp}$  19 \| end  20 end  21 $P^{w}=E_{sp}^{w}P^{sp}$  22 return $P^{w}$ |
