# Supplementary material for: Kinematic and aerodynamic modeling of flexible wings with wing root adjustment for flapping wing micro aerial vehicles
Source: Sci Rep. 2026 Mar 2;16:9827. doi: 10.1038/s41598-026-40582-8 (PMC13018484; doi:10.1038/s41598-026-40582-8)
Supplement: Supplementary file 1 — Supplementary Information. [file 41598_2026_40582_MOESM1_ESM.zip › supplementary/Algorithm 2.docx]

Algorithm 2: Aerodynamic Force Calculation for Discrete Rigid Wing Elements

The corresponding calculation process is shown in Algorithm 2: This algorithm takes the wing point movement trajectory output by Algorithm 1 as input and is specifically designed for calculating the aerodynamic forces of rigid wing elements. Its core calculation modules include solving the blade element length, angle of attack, and lift-drag coefficients, then calculating the aerodynamic forces by region and returning the simulation results.

| Algorithm 2: Aerodynamic calculation of discretized rigid wing surface elements |
| --- |
| Input:$P^{w}$,$P^{sp}$,f, Slices_step  Output: The aerodynamic effect of the wings  1 area division  2 if $P_{0,z}^{w}-P_{4,z}^{w}<-2*Slices_{s}\mathrm{tep}$  3 \| if $P_{2,z}^{w}-P_{1,z}^{w}>2*Slices_{s}\mathrm{tep}$  4 \| \| shape $⟵$ 1  5 \| else  6 \| \| shape $⟵$ 2  7 elseif $P_{0,z}^{w}-P_{4,z}^{w}>2*Slices_{s}\mathrm{tep}$  8 \| if $P_{2,z}^{w}-P_{1,z}^{w}>2*Slices_{s}\mathrm{tep}$  9 \| \| shape $⟵$ 3  10 \| else  11 \| \| shape $⟵$ 4  12 else  13 \| if $P_{2,z}^{w}-P_{1,z}^{w}>2*Slices_{s}\mathrm{tep}$  14 \| \| shape $⟵$ 5  15 \| else  16 \| \| shape $⟵$ 6  17 end  18 be_len $⟵$ blade_element_length_calculation(shape,$P^{w}$)  19 be_angle $⟵$ blade_element_attack_anglec_cal (shape,$P^{w}$)  20 $\left[ C_{N},C_{T},C_{L},C_{D} \right]$ $⟵$ aero_coefficient_cal(be_angle)  21 $\left[ F_{T},F_{D} \right]$ $⟵$ aero_cal(shape,$P^{w}$,$\left[ C_{L},C_{D} \right]$, be_angle, be_len)  22 return $\backslash left[F\_T,F\_D\backslash right]$ |
