# Supplementary figures and images for: Kinematic and aerodynamic modeling of flexible wings with wing root adjustment for flapping wing micro aerial vehicles
Source: Sci Rep. 2026 Mar 2;16:9827. doi: 10.1038/s41598-026-40582-8 (PMC13018484; doi:10.1038/s41598-026-40582-8)

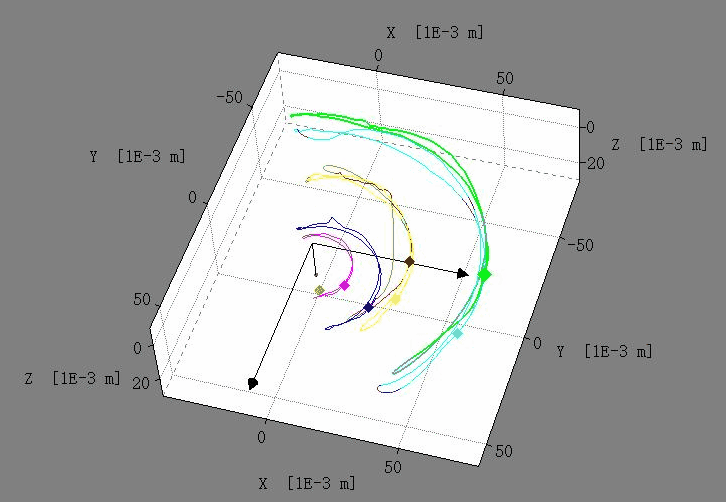

Supplement: Supplementary file 1 — Supplementary Information. [file 41598_2026_40582_MOESM1_ESM.zip › supplementary/supplementary video1.gif]
